# Synergistic Anti-Tumor Effects of Sulfatinib and Kaempferol on Pancreatic Neuroendocrine Tumors via CALCA-mediated PI3K/AKT/mTOR Pathway

**DOI:** 10.7150/ijbs.119176

**Published:** 2025-10-27

**Authors:** Lingyi Chen, Pengfei Liu, Fengjuan Chen, Bingyan Xue, Xu Han, Lijun Yan, Jianan Bai, Xiaoya Li, Min Liu, Ye Tian, Mujie Ye, Qiyun Tang

**Affiliations:** 1 Department of Neuroendocrine Tumor, The First Affiliated Hospital with Nanjing Medical University, Jiangsu, China.; 2 Department of Gastroenterology, Jiangyin People's Hospital, Jiangsu, China.; 3 Department of Gastroenterology, Xishan People's Hospital, Jiangsu, China.

**Keywords:** pancreatic neuroendocrine tumors, sulfatinib, kaempferol, pharmacotherapy

## Abstract

Pancreatic neuroendocrine tumors (pNETs) represent a diverse category of neoplasms originating from pancreatic neuroendocrine cells. Although these tumors generally exhibit a relatively indolent nature, they often metastasize early in their course, significantly affecting patient outcomes. Sulfatinib (SULF) is associated with considerable toxicity and resistance challenges, leading to many patients failing to achieve long-term disease management. In contrast, Kaempferol (KMP), a naturally occurring phytochemical, has shown considerable promise in anti-tumor treatments. Our study revealed that the combination of SULF and low-dose KMP enhances the sensitivity of pNET cells to SULF. Moreover, this combination demonstrated a synergistic effect on angiogenesis inhibition, observed in both *in vitro* and *in vivo* environments. Additionally, we confirmed this synergistic anti-tumor effect using a subcutaneous tumor model of pNETs. Transcriptome sequencing identified CALCA as a key molecule in the synergistic inhibition of pNETs proliferation by SULF and KMP. In summary, our findings provide novel insights into combination therapy for pNETs while elucidating the mechanistic role of CALCA in the modulation of angiogenesis. This research establishes a foundation for the development of vascular-targeted combination therapeutic strategies for the treatment of pNETs.

## Introduction

Pancreatic neuroendocrine tumors (pNETs) are an important subtype of gastroenteropancreatic neuroendocrine tumors (GEP-NETs), the incidence of which has increased in recent years^1^-^4^. Despite the small size of most primary pNETs lesions, they have a high propensity to metastasis at an early stage. This specific biological behavior leads to distant metastasis in 40-60% of patients at the time of diagnosis^5^. For limited pNETs (e.g. stage T1N0M0), surgical resection remains the preferred radical treatment option^6^, ^7^. However, conventional chemotherapy, biological therapy and targeted therapy have become important treatment options for advanced cases^8^-^11^.

A recent development from China, Sulfatinib is a novel oral small molecule tyrosine kinase inhibitor (TKI) that has been found to target vascular endothelial growth factor receptor1-3 (VEGFR1-3), fibroblast growth factor 1 (FGFR1), and colony-stimulating factor 1 receptor (CSF-1R). It has been shown to have a dual mechanism of anti-angiogenesis combined with immune microenvironment modulation, resulting in synergistic antitumor effects. Sulfatinib has been shown to be significantly effective in treating pNETs and extra pancreatic neuroendocrine tumors. Clinical studies have demonstrated the efficacy of Sulfatinib in treating pNETs^10^, ^12^, ^13^. However, the adverse effects of Sulfatinib in clinical applications, including hypertension, proteinuria, and diarrhea, should not be ignored^14^, ^15^. Therefore, there is an urgent need to study a novel dosing regimen that reduces the dosage of Sulfatinib and improves efficacy and safety.

Kaempferol, a dietary polyphenol flavonoid that is widely found in plants, has attracted much attention in the field of oncology as it exhibits significant antiproliferative and antiangiogenic properties^16^-^18^. Studies have shown that Kaempferol, when used in combination with chemotherapeutic agents, can significantly inhibit tumor cells proliferation and metastasis. This is achieved by down-regulating the activation of the PI3K/AKT/mTOR signaling pathway, inhibiting the downstream vascular endothelial growth factor (VEGF) and VEGFR2, and enhancing tumor cell sensitivity to chemotherapeutic agents. This produces a synergistic effect in chemotherapy^19^, ^20^. Furthermore, Kaempferol has been found to reduce the metastatic potential of tumor cells by inhibiting the secretion of inflammatory factors associated with metastasis^7^. However, the exact mechanism by which Kaempferol inhibits pancreatic neuroendocrine tumorigenesis and progression is not fully understood.

The aim of this study was to investigate whether the combination of Sulfatinib and Kaempferol synergistically inhibits the progression of pNETs. *In vitro* and *in vivo* models were used to determine the concentrations of the drug combination and to reveal the specific molecular mechanisms in which the highly expressed CALCA gene in the combination group may be involved, as well as its potential as a therapeutic target or biomarker. This will provide valuable insights that can be used to develop more effective targeted therapies and individualized medicine.

## Methods

### Chemicals and reagents

Sulfatinib and Kaempferol were purchased from Selleck Chemicals (S0487, HMPL-012, China) and MedChemExpress (HY-14590, Robigenin, Shanghai, China), respectively. Both compounds were prepared as stock solutions in dimethyl sulfoxide (DMSO) and stored at ultra-low temperatures until use.

### Cell culture and treatment

The human neuroendocrine tumors (NETs) cell lines BON-1 and QGP-1 were utilized. BON-1 cells (derived from human pNETs) were provided by Professor Xianjun Yu of Fudan University Shanghai Cancer Center. QGP-1 cells (another human pNETs line) and human umbilical vein endothelial cells (HUVECs) were sourced from the JCRB (JCRB0183) and ATCC (CRL-1730), respectively. Cells were propagated in their respective media: BON-1 and HUVECs in DMEM/F-12 (1:1, Gibco), and QGP-1 in RPMI-1640 (Gibco). All culture media contained 10% fetal bovine serum (FBS,Yeasen Biotechnology, Shanghai, China) and 1% penicillin-streptomycin (New Cell & Molecular Biotech, Suzhou, China), and cells were grown at 37°C in a humidified 5% CO₂ incubator.

### Cytotoxicity assays

To assess cytotoxicity, BON-1 and QGP-1 cells were plated in 96-well plates at a density of 5 × 10³ cells per well and allowed to adhere. Subsequently, the cells were exposed to a concentration gradient of Sulfatinib or Kaempferol, prepared by serial dilution in their respective complete culture media. Following treatment, cell viability was determined by the CCK-8 assay (New Cell & Molecular Biotech). Specifically, a solution of complete medium and CCK-8 reagent (1:10) was added to the wells, and the plates were incubated for 1 hour. The optical density (OD) at 450 nm was then measured with a microplate reader. The half-maximal inhibitory concentration (IC50) was computed from the dose-response data using GraphPad Prism 9.0 (GraphPad, La Jolla, CA, USA).

### Synergy determination with SynergyFinder

BON-1 and QGP-1 cells were plated in 96-well plates at 5 × 10³ cells per well and treated with Sulfatinib, Kaempferol, or their combination, based on the concentration ranges defined in the preceding cytotoxicity assay. The concentrations tested for Kaempferol included 50 μM, 100 μM, 200 μM, 400 μM, and 800 μM. In contrast, the concentrations for Sulfatinib comprised 12.5 μM, 25 μM, 50 μM, 100 μM, and 200 μM. After 24-hour of drug exposure, cell viability was assessed using the CCK-8 assay. Drug interaction was analyzed via the Zero Interaction Potency (ZIP) model using the SynergyFinder web tool (https://synergyfinder.fimm.fi). The Inhibition Index (100 - viability) was used as the response metric^21^. ZIP scores exceeding 0 and surpassing 10 were interpreted as synergistic and strongly synergistic, respectively^22^. A response heatmap was generated to visualize the therapeutic potential of the combinations.

### Colony formation assays

A colony formation assay was performed by plating 1 × 10⁴ BON-1 and QGP-1 cells per well of a 6-well plate. The cells were maintained in culture with 10% FBS-supplemented medium for 12 days under standard incubator conditions. Following the incubation period, the colonies were fixed with 4% paraformaldehyde for 30 minutes and then stained with 0.2% crystal violet for an additional 30 minutes. Quantification of colonies was carried out using ImageJ software.

### Cell apoptosis assessment

Cell apoptosis under different treatment conditions was evaluated by flow cytometry with an apoptosis detection kit (AP101, LIANKE Biotech, Hangzhou, China). Briefly, post-treatment cells were digested with EDTA-free trypsin (C100C1, New Cell & Molecular Biotech, Suzhou, China) and collected. The cell pellets were then resuspended in 500 μL of Annexin binding buffer. For staining, a working solution containing both 5 μL of Annexin V-FITC and 5 μL of propidium iodide (PI) was added to the cell suspension, which was incubated for 30 minutes in the dark. Finally, the stained cells were analyzed on a BD FACSVerse flow cytometer (BD Bioscience, USA), and the data were processed using FlowJo 10.6 software.

### Wound-healing assays

HUVECs were preconditioned by co-culture in a medium that facilitated the expression or suppression of BON-1. The cells were plated in 6-well plates at 2 × 10^6^ cells per well. Once a confluent monolayer formed, two straight wounds were introduced per well using a pipette tip. The spent medium was then aspirated and replaced with fresh serum-free medium. Photographic documentation of the wound areas was performed at 0 and 24 hours under a light microscope. The resulting images were analyzed with ImageJ software to determine the cell migration distance.

### Angiogenesis experiment

To induce quiescence, HUVECs were incubated in DMEM supplemented with 0.2% FBS for 24 hours. Meanwhile, a pre-chilled 24-well plate was coated with 50 µM of thawed Matrigel matrix, maintaining sterility and keeping all components on ice. The HUVECs were then trypsinized, resuspended, and seeded onto the gel at 3 × 10⁴ cells per well. The plate was placed in a humidified incubator at 37°C and 5% CO₂. Tube formation was documented at 4-6-hour intervals by photomicrography under standardized exposure and contrast conditions. The resulting images were analyzed for tubule length and network complexity using the Angiogenesis Analyzer tool in ImageJ.

### Western blot analysis

Cells were lysed using ice-cold NP40 lysis buffer (Beyotime, Nantong, China), which was supplemented with the protease inhibitor phenylmethylsulfonyl fluoride (Beyotime). Protein concentrations were determined by the Bradford method using a Coomassie Brilliant Blue G250 kit (Beyotime). After denaturing the proteins at 95 °C for 10 minutes, equal amounts of protein were separated by SDS-PAGE (New Cell & Molecular Biotech, Suzhou, China) and transferred electrophoretically onto polyvinylidene fluoride (PVDF) membranes. The membranes were blocked with 8% skimmed milk for one hour, followed by incubation with primary antibodies at 4 °C overnight. After incubation with primary antibodies overnight at 4°C, the membranes were washed three times with TBST. They were then incubated with horseradish peroxidase (HRP)-conjugated secondary antibodies (diluted at 1:5000) for 1 hour at room temperature. After extensive washing, the protein bands were visualized using an enhanced chemiluminescence (ECL) detection kit. GAPDH was used as a loading control to ensure equal protein loading. All antibodies used are listed in Supplementary Table S1. The anti-NBASP polyclonal antibody was raised in rabbits and supplied by Hangzhou Hua'an Technology (Hangzhou, China).

### RNA extraction and quantitative real-time PCR (qPCR)

Total RNA was isolated from cells with Trizol reagent (Vazyme, Nanjing, China), and its concentration was measured using a Nanodrop 2000 spectrophotometer. The extracted RNA was then reverse-transcribed into complementary DNA (cDNA) with the 4× Hifair® III SuperMix plus (Yeasen), in accordance with the manufacturer's protocol. Real-time PCR was subsequently performed with the ChamQ Universal SYBR qPCR Master Mix. All primer sequences used are provided in Table S2. RNA sequencing (RNA-SEq), using Trizol® (Takara Bio, Inc.) to isolate total RNA from BON-1 and QGP-1 cells treated with drugs for 24 hours and the corresponding control groups. The obtained RNA samples were sequenced by Hangzhou Lianchuan Biotechnology Co., LTD.

### Construction of animal models

Four- to six-week-old male BALB/c nude mice were inoculated subcutaneously in both axillary regions with 100 µL of BON-1 cell suspensions (5 × 10⁶ cells) overexpressing CALCA, CALCA knockout cells, or negative control cells, delivered in phosphate-buffered saline (PBS). The animals were maintained in plastic cages fitted with filter caps under laminar flow ventilation. To assess the inhibitory effects of Sulfatinib and Kaempferol on tumor growth, the mice were randomized into four groups: PBS control, Sulfatinib alone, Kaempferol alone, and Sulfatinib combined with Kaempferol. Intraperitoneal administration of 0.12 mg/kg Sulfatinib and/or 1 mg/kg Kaempferol was performed every two days. After three weeks, all mice were euthanized, and the tumors were harvested for measurement and imaging. Tumor tissues were either fixed in 4% paraformaldehyde for subsequent histology or frozen at -80°C for further analysis. Tumor volume was determined using the formula: Volume (mm³) = length × width² × 0.5. Immunohistochemical and immunofluorescence staining were subsequently conducted on both visceral and tumor tissue sections. All animal experiments were approved by the Institutional Animal Care and Use Committee (IACUC) of Nanjing Medical University.

### Statistics analysis

All statistical analyses were performed using Prism 9.0 (GraphPad, La Jolla, USA) and the online SynergyFinder platform. Data are presented as mean ± standard deviation (SD). All experiments were conducted with three independent replicates. Differences between groups were evaluated using appropriate statistical tests, including Student's t-test, unpaired t-test, One-way ANOVA and Two-way ANOVA, following verification of variance homogeneity. Statistical significance was defined as *P < 0.05, **P < 0.01, ***P < 0.001, ****P < 0.0001 and ns: no significance.

## Results

### Kaempferol synergistically enhances the cytotoxicity of Sulfatinib on pNETs cells

**Figure 1:**
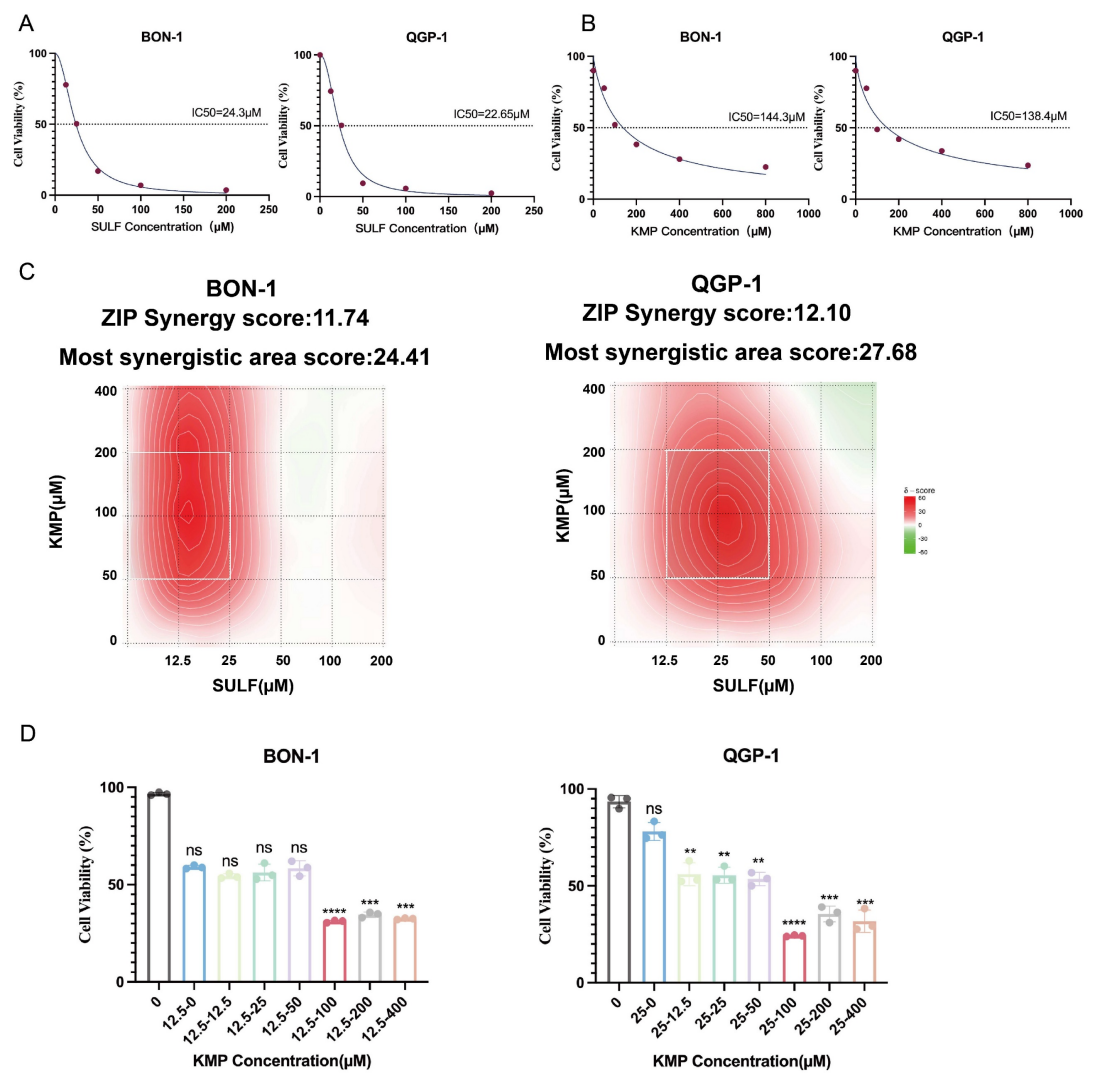
Kaempferol synergistically enhances the cytotoxicity of Sulfatinib on pNET cells. (A) A dose-response study has been conducted on Sulfatinib in BON-1 and QGP-1 cells, with the resultant IC50 values. (Data were shown as mean±SD. n=3. The curve was fitted using a four-parameter logistic model (nonlinear regression)). (B) A similar approach has been adopted for a dose-response study of Kaempferol in BON-1 and QGP-1 cells, with the corresponding IC50 values. (Data were shown as mean±SD. n=3. The curve was fitted using a four-parameter logistic model (nonlinear regression)). (C) The following heatmap illustrates the drug combination: The present study investigates the synergistic effects of Sulfatinib and Kaempferol on BON-1 and QGP-1 cells. The cells were exposed to a concentration gradient for a period of 24 hours, after which the cell viability was evaluated using a CCK-8 assay. This assessment was based on the IC50 value that had been previously determined for the two drugs mentioned above. The ZIP synergy score was calculated using Synergyfinder software. A score greater than 0 indicates synergy, and a score greater than 10 indicates strong synergy. The gradient of the red area is indicative of the strength of synergy. The white rectangle indicates the concentration at which the drug combination had the strongest synergy, and the corresponding X and Y axes on either side of the white rectangle indicate the concentration at which the drug combination had the greatest inhibitory effect on cell growth. (D) In order to ascertain the most efficacious dose of Kaempferol when administered concomitantly with Sulfatinib, viability assays were conducted on BON-1 and QGP-1 cells utilizing various proportional dosing regimens. (Data were shown as mean±SD. n=3. Statistical significance between groups was determined by one-way ANOVA). *p < 0.05, **p < 0.01, ***p < 0.001, ****p < 0.0001, ns: no significance.

The current investigation aimed to elucidate the pharmacological impacts of Sulfatinib and Kaempferol on pancreatic neuroendocrine tumors (pNETs) cells. To achieve this objective, the biological effects of Sulfatinib were evaluated in the pNETs cell lines BON-1 and QGP-1 through the use of the CCK-8 assay, from which dose-response curves were generated (Fig. 1A). Furthermore, the cytotoxicity of Kaempferol was assessed utilizing the same methodology, allowing for the calculation of its IC50 values (Fig. 1B). The findings indicated that both Sulfatinib and Kaempferol displayed concentration-dependent antiproliferative properties. The ZIP synergy scores were computed based on the IC50 values and concentration gradients of the two compounds, employing the online SynergyFinder software (Fig. 1C). The analysis revealed that the average (and peak) proportion of antitumor effects resulting from drug-drug interactions was 11.74 (24.41) for BON-1 cells and 12.10 (27.68) for QGP-1 cells, respectively. Notably, Sulfatinib and Kaempferol exhibited a robust synergistic effect in suppressing tumor proliferation (ZIP synergy score > 10), with the white rectangle denoting the area of maximum synergy (Fig. 1C). The results further indicated that the optimal synergistic concentrations of Sulfatinib were 12.5 μM and 25 μM in BON-1 and QGP-1 cells, respectively, both of which fell within the lower effective range of the maximum synergy region. Consequently, Kaempferol demonstrated the most significant synergistic antiproliferative effect at a concentration of 100 μM (Fig. 1D).

### Sulfatinib combined with Kaempferol inhibits tumor proliferation, angiogenesis, and increase apoptosis

**Figure 2:**
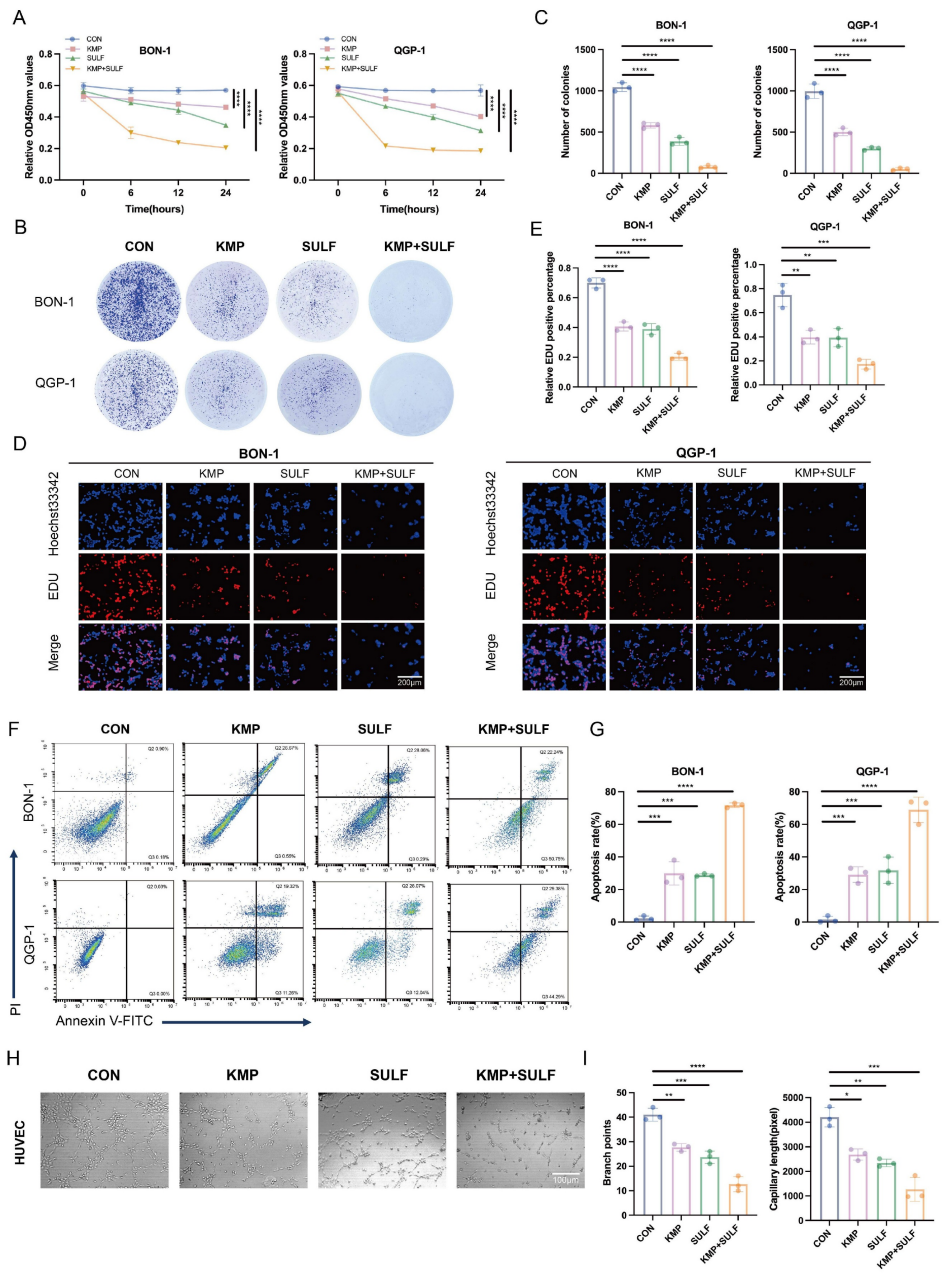
Sulfatinib combined with Kaempferol inhibits tumor proliferation, angiogenesis, and increase apoptosis. (A-G) We detected the proliferation and apoptosis of BON-1 and QGP-1 cells treated with Sulfatinib, Kaempferol and combination therapy for 24 hours using the following methods: (A) CCK-8 assay, (B,C) colony formation assay, (D,E) EDU assay and (F,G) apoptosis. (Data were shown as mean±SD. n=3. Statistical significance between groups was determined by one-way ANOVA). Scale bar=200μm. (H,I) We detected tubule formation in Sulfatinib, Kaempferol and combination therapy-treated HUVEC cells for 24 hours. (Data were shown as mean±SD. n=3. Statistical significance between groups was determined by one-way ANOVA). Scale bar=100μm. *p < 0.05, **p < 0.01, ***p < 0.001, ****p < 0.0001, ns: no significance.

In this study, we explored the possible synergistic anti-tumor effects of Sulfatinib and Kaempferol in pNETs. We assessed their influence on tumor cell proliferation, apoptosis, and angiogenesis *in vitro* utilizing BON-1 and QGP-1 pNETs cell lines, along with HUVECs. The combination of Sulfatinib and Kaempferol exhibited a significant and marked reduction in the proliferation of BON-1 and QGP-1 cells when compared to the effects of each drug administered separately. The results from the CCK-8 assay indicated that both BON-1 and QGP-1 cells treated with KMP and SULF for 24 hours displayed decreased growth rates (Fig. 2A). Furthermore, colony formation assays reinforced these findings, revealing that the combination treatment led to fewer and smaller colonies relative to the single-agent treatment groups (Fig. 2B,C). EdU assays demonstrated a notable decrease in DNA synthesis in the combination treatment group (Fig. 2D,E), suggesting a compromised capacity for cell proliferation. Flow cytometry analyses revealed that the combination of Sulfatinib and Kaempferol resulted in a significant rise in the number of apoptotic cells compared to the administration of each agent alone (Fig. 2F,G). This observation indicates that the combination therapy not only inhibited tumor cell growth but also actively facilitated their apoptosis. Given that Sulfatinib targets VEGF signaling, this study also assessed whether the combination of KMP and SULF enhances its anti-angiogenic properties by evaluating branch points and capillary length through angiogenesis assays. The experiments demonstrated that the combination was more effective at disrupting endothelial network formation than either agent used individually (Fig. 2H,I), suggesting that the dual blockade of Sulfatinib targeting the VEGFR and the multi-targeting capabilities of Kaempferol work synergistically to inhibit angiogenesis. Consequently, the results obtained indicate that the concurrent use of KMP and SULF in pNETs cell lines leads to a synergistic inhibition of both cell proliferation and angiogenesis *in vitro*.

### Sulfatinib combined with Kaempferol enhances tumor growth inhibition *in vivo*

**Figure 3:**
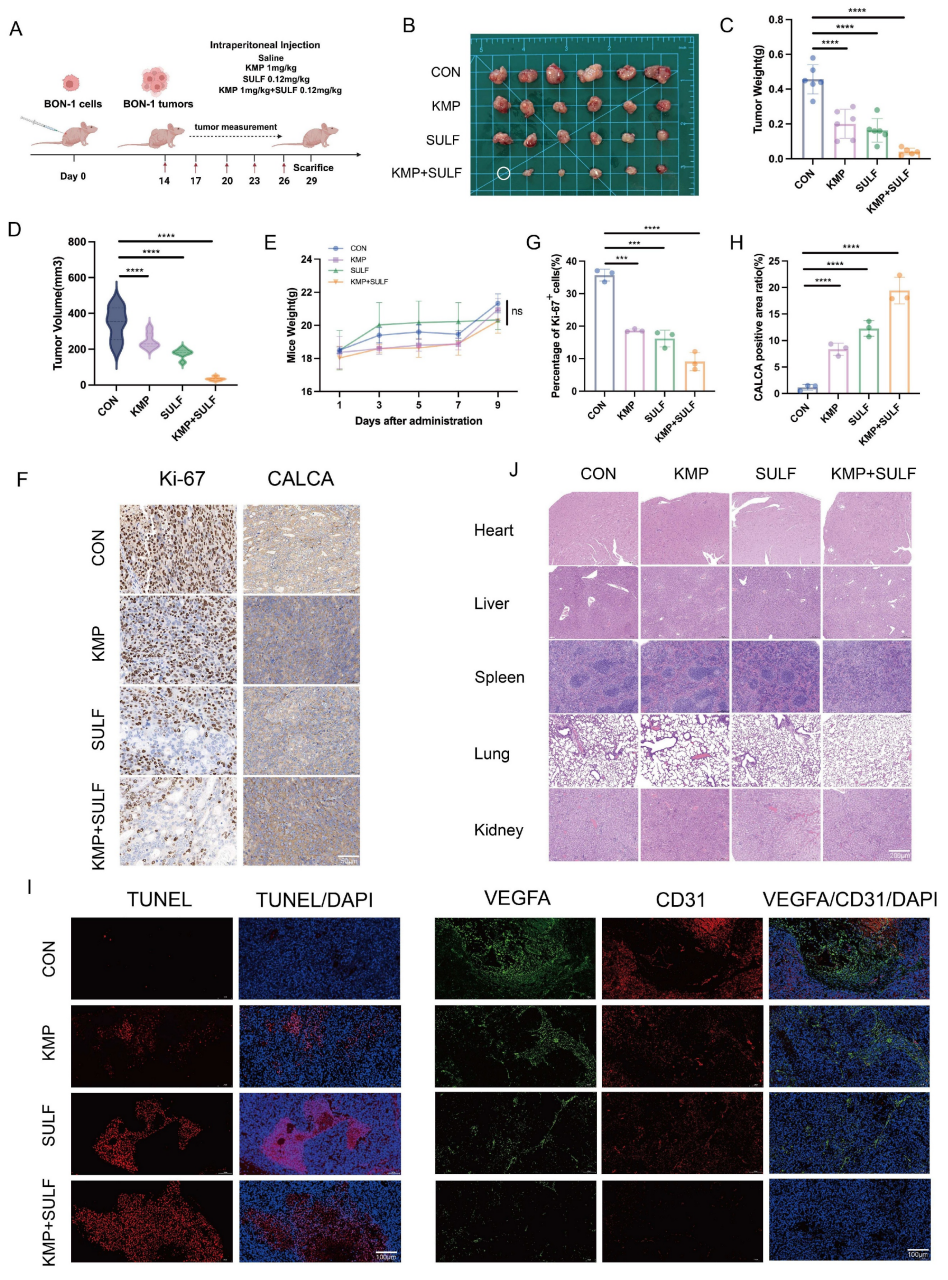
Sulfatinib combined with Kaempferol enhances tumor growth inhibition *in vivo*. (A) The following is the model of nude mice drug administration protocol. (B)Images of subcutaneous tumors removed from nude mice. (C-E) Tumor weight, tumor volume, and nude mice body weight of different groups of nude mice. (Data were shown as mean±SD. n=6. Statistical significance between groups was determined by one-way ANOVA). (F-H) The images show representative IHC staining of Ki-67 and CALCA, along with quantitative analysis. (Data were shown as mean±SD. n=3. Statistical significance between groups was determined by one-way ANOVA). Scale bar=50μm. (I) Immunofluorescence apoptosis staining of tumor tissues by TUNEL and angiogenesis-associated staining by VEGFA, CD31. Scale bar=100μm. (J) HE staining of nude mouse organs from various groups of nude mice. Scale bar=200μm. The *in vivo* study detected no toxicity. *p < 0.05, **p < 0.01, ***p < 0.001, ****p < 0.0001, ns: no significance.

To evaluate the combined antitumor effectiveness of Sulfatinib and Kaempferol against pNETs, we developed a xenograft mouse model utilizing BON-1 cells. We examined various parameters including tumor growth, apoptosis, angiogenesis, and the impact of drug treatment on systemic toxicity. The purpose of this investigation was to explore the synergistic effects of Sulfatinib and Kaempferol *in vivo*. For this study, a subcutaneous tumor model was created in nude mice through the implantation of BON-1 cells (Fig. 3A). After a duration of three weeks, intraperitoneal injections were administered to the mice across four groups: saline control, Kaempferol alone (1 mg/kg), Sulfatinib alone (0.12 mg/kg), and a combination of Kaempferol (1 mg/kg) and Sulfatinib (0.12 mg/kg). At the conclusion of the experimental period, the mice were euthanized, and tumor images were captured (Fig. 3B). The combination of Sulfatinib and Kaempferol demonstrated enhanced antitumor effects compared to monotherapy *in vivo*. Notably, the combination therapy group exhibited a significant reduction in both tumor weight and volume when compared to the monotherapy and control groups (Fig. 3C,D). Additionally, the safety profile of the combination therapy was deemed acceptable, as indicated by the absence of considerable weight loss or organ toxicity (Fig. 3.E,J). Further immunohistochemical (IHC) analyses supported this mechanism of action: Ki-67 staining illustrated a significant reduction in proliferating cells within the combination treatment group (Fig. 3F,G), aligning with the findings from the *in vitro* studies. Immunofluorescence (IF) analysis revealed that the combination therapy significantly enhanced apoptosis and inhibited tumor angiogenesis in pNETs relative to the individual treatments. TUNEL staining indicated that the two-drug combination led to increased apoptosis levels among tumor cells. In contrast, staining for the endothelial markers VEGFA and CD31 showed a decrease in microvessel density, effectively suppressing angiogenesis (Fig. 3I). This observation is consistent with the outcomes of the *in vitro* angiogenesis assay, reinforcing the dual inhibitory effect of Sulfatinib and Kaempferol on angiogenesis.

### Transcriptome sequencing reveals high expression of CALCA in the co-drug group

**Figure 4:**
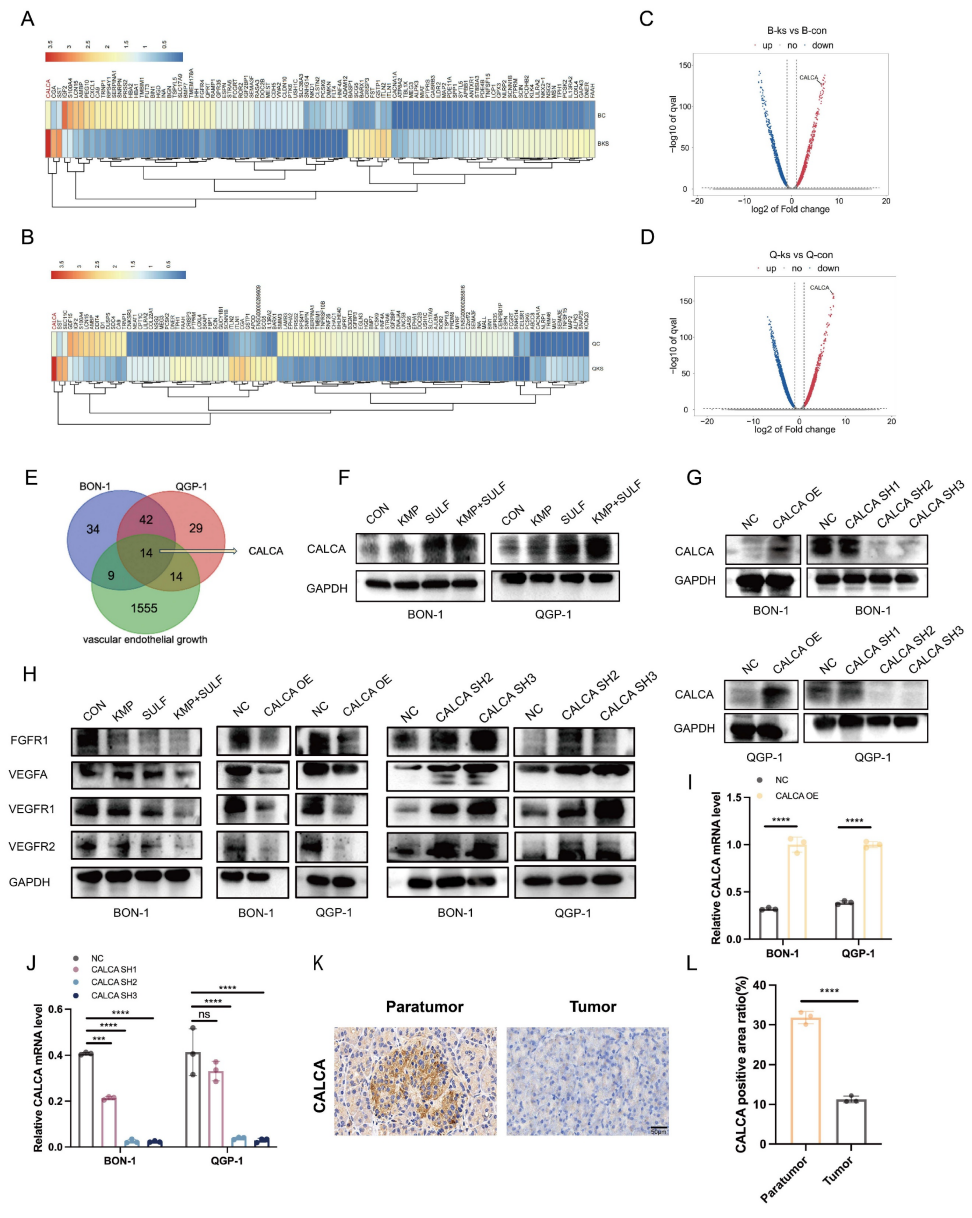
Transcriptome sequencing reveals high expression of CALCA in the co-drug group. (A-D) The heatmap illustrates the differential expression of CALCA in the control and combination groups. (E) The Venn diagram shows the overlapping genes identified by RNA-seq analysis. (F)Western blot analysis of the protein level of CALCA in Sulfatinib alone, Kaempferol alone, and combined therapy of Sulfatinib and Kaempferol, as well as control groups. (G) CALCA protein expression in BON-1 cells and QGP-1 cells overexpressing and knockdown of CALCA. (H) Expression of angiogenesis-related proteins in CALCA-manipulated stable cell lines and following drug treatments. (I,J) The mRNA levels of CALCA knockdown and over-expression in BON-1 cells and QGP-1 cells. (Data were shown as mean±SD. n=3. Statistical significance between groups was determined by two-way ANOVA). (K,L) Immunohistochemistry of the expression of CALCA in pNETs and normal peritumor tissues. (Data were shown as mean±SD. n=3. Statistical significance between groups was determined by two-tailed unpaired Student's t-test). Scale bar=50μm. *p < 0.05, **p < 0.01, ***p < 0.001, ****p < 0.0001, ns: no significance.

Transcriptome sequencing has been employed to elucidate CALCA as a pivotal mediator of drug synergy. To ascertain the molecular targets responsible for the synergistic anti-tumor effects associated with Sulfatinib and Kaempferol, transcriptome sequencing analysis was conducted on pNETs cells subjected to treatment. A subsequent analysis through heatmap visualization indicated a significant variation in CALCA expression levels between the experimental group and the control group (Fig. 4A-D). Venn diagram illustrated that CALCA emerged as one of the fourteen fundamental genes derived from the intersection of angiogenesis-related genes with differentially expressed genes from drug-treated BON-1 and QGP-1 cells (Fig. 4E). Validation via Western blotting further confirmed an increase in the protein levels of CALCA in both drug-treated and untreated wild-type groups, with notably higher expression observed in the co-drug group (Fig. 4F). In the next phase of the study, stable cell lines were developed to facilitate both the expression and knockdown of CALCA through lentiviral transfection. The efficiency of CALCA expression and knockdown was subsequently validated by Western blot analysis in the BON-1 and QGP-1 NET cell lines (Fig. 4G). Additionally, qRT-PCR validation was executed (Fig. 4.I,J). To assess CALCA expression in patients diagnosed with pNETs, IHC staining was conducted on tumor tissues and adjacent normal pancreatic tissues from these individuals. The findings revealed that CALCA levels were significantly elevated in normal pancreatic tissues compared to tumor tissues (Fig. 4K,L). Furthermore, IHC staining for CALCA was performed in the *in vivo* experiments conducted earlier, which indicated that the highest expression levels were found in the combination treatment group (Fig. 3F,H). An investigation into the interplay between Sulfatinib, Kaempferol, and CALCA, along with key targets of tumor angiogenesis, was also undertaken. Western blot analysis corroborated that the combination therapy markedly diminished tumor angiogenesis relative to both the control group and monotherapy. Moreover, the overexpression of CALCA significantly inhibited angiogenesis, whereas CALCA knockdown facilitated vascular neogenesis, subsequently accelerating tumor progression (Fig. 4H). The comprehensive data derived from these experiments indicate that CALCA serves as a crucial regulatory gene in the context of combination therapy, with its expression levels exhibiting a significant correlation to anti-tumor efficacy. Further investigations will aim to substantiate the role of CALCA in combination therapy within both *in vitro* and *in vivo* experimental models.

### CALCA inhibits proliferation, apoptosis, and angiogenesis in pNETs cells

**Figure 5:**
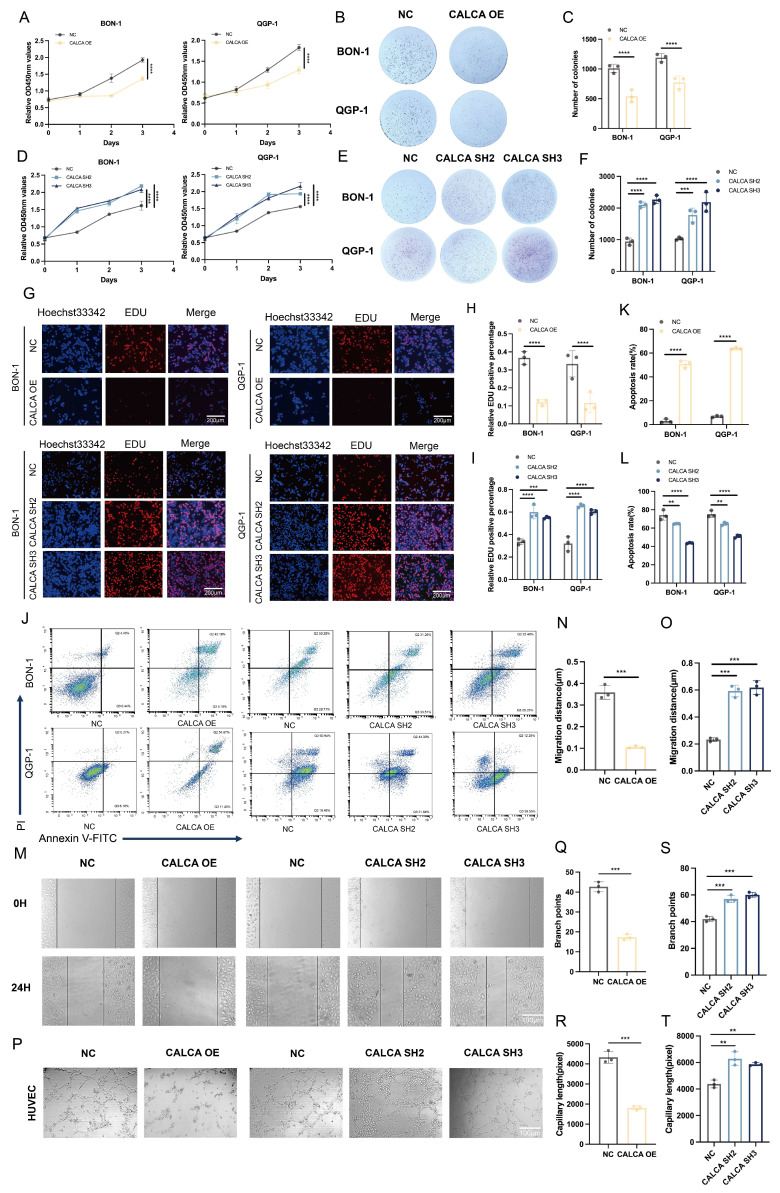
Altered CALCA expression affects proliferation, apoptosis, and angiogenesis in pNET cells. (A)CCK-8 assay was performed in BON-1 and QGP-1 cells with negative control or CALCA overexpression. (Data were shown as mean±SD. n=3. Statistical significance between groups was determined by two-tailed unpaired Student's t-test). (B-C) Colony formation assay was performed in BON-1 and QGP-1 cells with negative control or CALCA overexpression. (Data were shown as mean±SD. n=3. Statistical significance between groups was determined by two-way ANOVA). (D)CCK-8 assay in BON-1 and QGP-1 cells with negative control or CALCA knockdown. (Data were shown as mean±SD. n=3. Statistical significance between groups was determined by one-way ANOVA). (E-F) Colony formation assay was performed in BON-1 and QGP-1 cells transfected with negative control or CALCA knockdown constructs. (Data were shown as mean±SD. n=3. Statistical significance between groups was determined by two-way ANOVA). (G-I) EDU assay was performed in BON-1 and QGP-1 cells with modulated CALCA expression. Scale bar = 200μm. (Data were shown as mean±SD. n=3. Statistical significance between groups was determined by two-way ANOVA). (J-L) Flow cytometry analysis demonstrated that modulating CALCA expression affects apoptosis in pNETs cells. (Data were shown as mean±SD. n=3. Statistical significance between groups was determined by two-way ANOVA). (M-O) A wound healing assay was performed using pNETs cells transfected with CALCA and co-cultured with HUVEC cells for 24 hours. Quantitative data for the CALCA overexpression group (N) are shown. (Data were shown as mean±SD. n=3. Statistical significance between groups was determined by two-tailed unpaired Student's t-test). Quantitative data for the CALCA knockdown group (O). (Data were shown as mean±SD. n=3. Statistical significance between groups was determined by one-way ANOVA). Scale bar = 100μm. (P-T) A tube formation assay was performed by co-culturing HUVEC cells with CALCA-transfected pNETs cells for 24 hours. Quantitative data for the CALCA overexpression (Q,R). (Data were shown as mean±SD. n=3. Statistical significance between groups was determined by two-tailed unpaired Student's t-test). Quantification of the CALCA knockdown group is shown in (S,T). (Data were shown as mean±SD. n=3. Statistical significance between groups was determined by one-way ANOVA). Scale bar = 100μm.*p < 0.05, **p < 0.01, ***p < 0.001, ****p < 0.0001, ns: no significance.

In a previous investigation, stable cell lines exhibiting either overexpression or knockdown of CALCA were successfully established through lentiviral transfection. The current study expands upon this foundation by conducting a series of *in vitro* phenotyping experiments aimed at elucidating the role of CALCA in pNETs cell lines, specifically with respect to its impact on tumor proliferation and angiogenic processes. The findings indicate that CALCA overexpression significantly inhibited both the proliferation and colony-forming abilities of BON-1 and QGP-1 cells, while CALCA knockdown yielded contrary results (Fig. 5A-F). An EdU incorporation assay further demonstrated that tumor cells with elevated CALCA levels exhibited a marked reduction in DNA replication, whereas CALCA knockdown cells displayed enhanced proliferative capacities (Fig. 5G-I). Flow cytometry analyses underscored the pivotal role of CALCA in promoting the survival of BON-1 and QGP-1 cells, revealing that CALCA expression was associated with a notable increase in apoptosis when compared to the negative control cohort. Additionally, to further delineate the role of the CALCA knockdown condition in apoptosis, the pro-apoptotic effects of Everolimus were utilized. It was observed that the CALCA knockdown group experienced a diminished apoptotic rate relative to the control group, which underwent the same treatment (Fig. 5J-L). Wound healing assays demonstrated that the migration of HUVECs was notably inhibited when co-cultured with CALCA-overexpressing pNETs cells, whereas CALCA knockdown significantly enhanced HUVECs migration (Fig. 5M-O). Furthermore, angiogenesis assays corroborated that CALCA overexpression markedly suppressed angiogenesis in co-culture systems, while CALCA knockdown resulted in an increase in both vascular branch points and angiogenesis length (Fig. 5P-T). Collectively, these results indicate that CALCA serves as a crucial regulator of cell survival in pNETs.

### CALCA acts as a tumor suppressor *in vivo*

**Figure 6:**
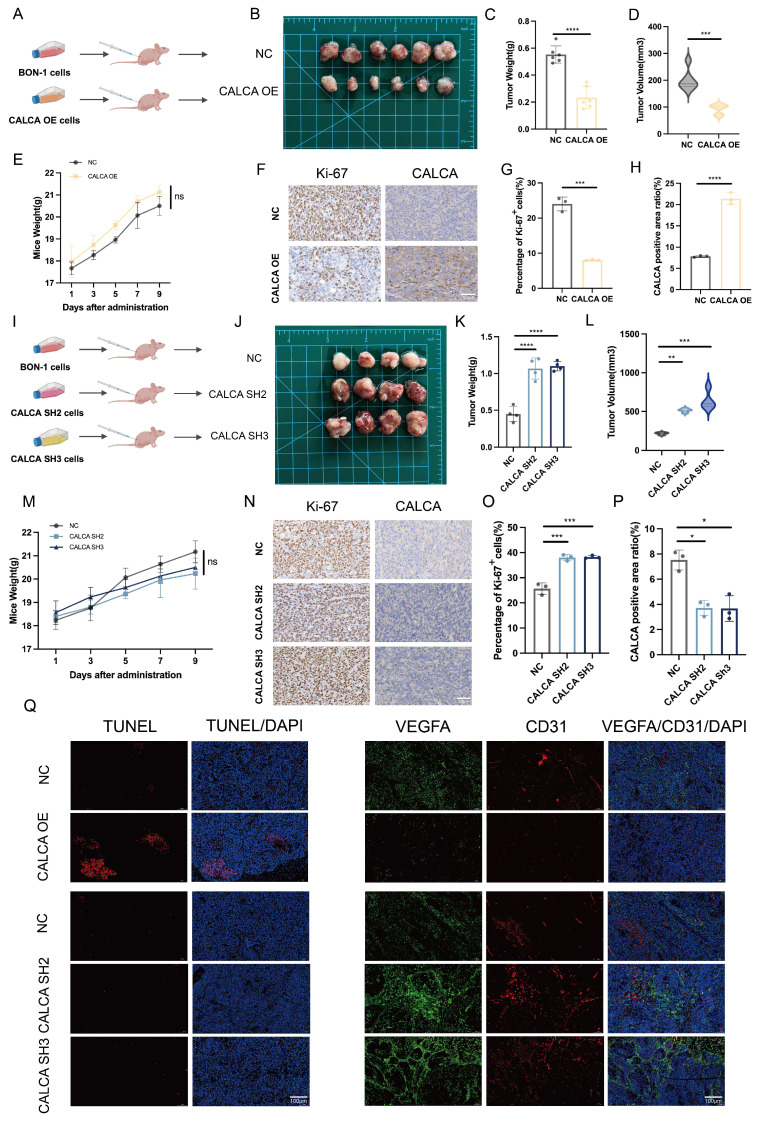
CALCA is a tumor growth inhibitor *in vivo*. (A-E) Primary tumor samples obtained after subcutaneous injection of cells overexpressing CALCA and control cells in nude mice. (A-B) Tumorigenic model and tumor images of nude mice injected subcutaneously. (C)Tumor weight, (D)tumor volume, and (E) nude mouse weight in both groups of nude mice. (Data were shown as mean±SD. n=6. Statistical significance between groups was determined by two-tailed unpaired Student's t-test). (F-H) Images show representative IHC staining and quantitative analysis of Ki-67 and CALCA. Primary tumor samples were taken from nude mice infected with CALCA knockdown BON-1 cells and controls by subcutaneous injection. (Data were shown as mean±SD. n=3. Statistical significance between groups was determined by two-tailed unpaired Student's t-test). Scale bar = 50μm. (I-J) Nude mouse subcutaneous tumorigenic model and tumor images. (K)Tumor weight, (L)tumor volume, and (M) nude mouse weight in both groups of nude mice. (Data were shown as mean±SD. n=4. Statistical significance between groups was determined by one-way ANOVA). (N-P) Images show the expression and quantitative analysis of KI-67 and CALCA in xenograft tumor tissues. (Data were shown as mean±SD. n=3. Statistical significance between groups was determined by one-way ANOVA). Scale bar = 50μm. (Q)Immunofluorescence, apoptosis TUNEL staining and angiogenesis-related VEGFA and CD31 staining of CALCA overexpression and knockdown tumor tissues. Scale bar = 100μm. *p < 0.05, **p < 0.01, ***p < 0.001, ****p < 0.0001, ns: no significance.

To elucidate the *in vivo* mechanism underlying the action of CALCA, BON-1 cells were engrafted into nude mice following infection with CALCA overexpression vectors. A xenograft model was successfully established, facilitating a systematic analysis of tumor growth characteristics and alterations in the tumor microenvironment (Fig. 6A). Notably, tumors in the CALCA overexpression group exhibited a significant reduction in both weight and volume, indicating a substantial inhibitory effect on tumor progression (Fig. 6B-D). Importantly, no statistically significant changes in the body weight of the nude mice were noted, suggesting that the intervention had minimal systemic toxicity (Fig. 6E). IHC staining for Ki-67 and CALCA demonstrated a negative regulatory relationship between CALCA expression and tumor tissue growth (Fig. 6F-H). In a parallel experiment, BON-1 cells engineered for CALCA knockdown were also implanted into nude mice (Fig. 6I). This modification led to a marked increase in tumor weight and volume, as well as an accelerated tumor progression in the knockdown group compared to controls (Fig. 6J-L). Again, body weight measurements of the nude mice remained stable (Fig. 6M). IHC analysis revealed a notable increase in Ki-67 expression levels within CALCA knockdown tumors, reinforcing the assertion of CALCA's role as a tumor suppressor (Fig. 6N-P). Furthermore, IF analysis indicated that tumors with stable CALCA overexpression exhibited heightened TUNEL staining and diminished endothelial markers VEGFA and CD31 in comparison to controls. This observation lends further credence to the hypothesis that CALCA expression fosters apoptosis while inhibiting angiogenesis in pNETs. Conversely, CALCA knockdown tumors displayed reduced TUNEL staining alongside a significant elevation in endothelial markers VEGFA and CD31, corroborating findings from the *in vitro* phenotyping assay (Fig. 6Q). In conclusion, these results underscore CALCA's role as a potent endogenous regulator of pNETs growth, achieved through the promotion of apoptotic pathways and the suppression of angiogenic processes. This mechanistic insight supports the observed synergistic effects between Sulfatinib and Kaempferol, thereby underscoring CALCA's potential as a valuable biomarker in therapeutic contexts.

### Sulfatinib and Kaempferol combination induces CALCA to inhibit malignant behavior of pNETs cells

The findings of the current investigation highlight that CALCA plays a pivotal role in inhibiting the malignant characteristics associated with pNETs. To determine whether the dual treatment of Sulfatinib and Kaempferol could mitigate the aggressive phenotype manifested by CALCA knockdown in pNETs cell lines, a series of proliferation assays were conducted on BON-1 and QGP-1 cells that were either subjected to CALCA knockdown or received the aforementioned combined treatment.

**Figure 7:**
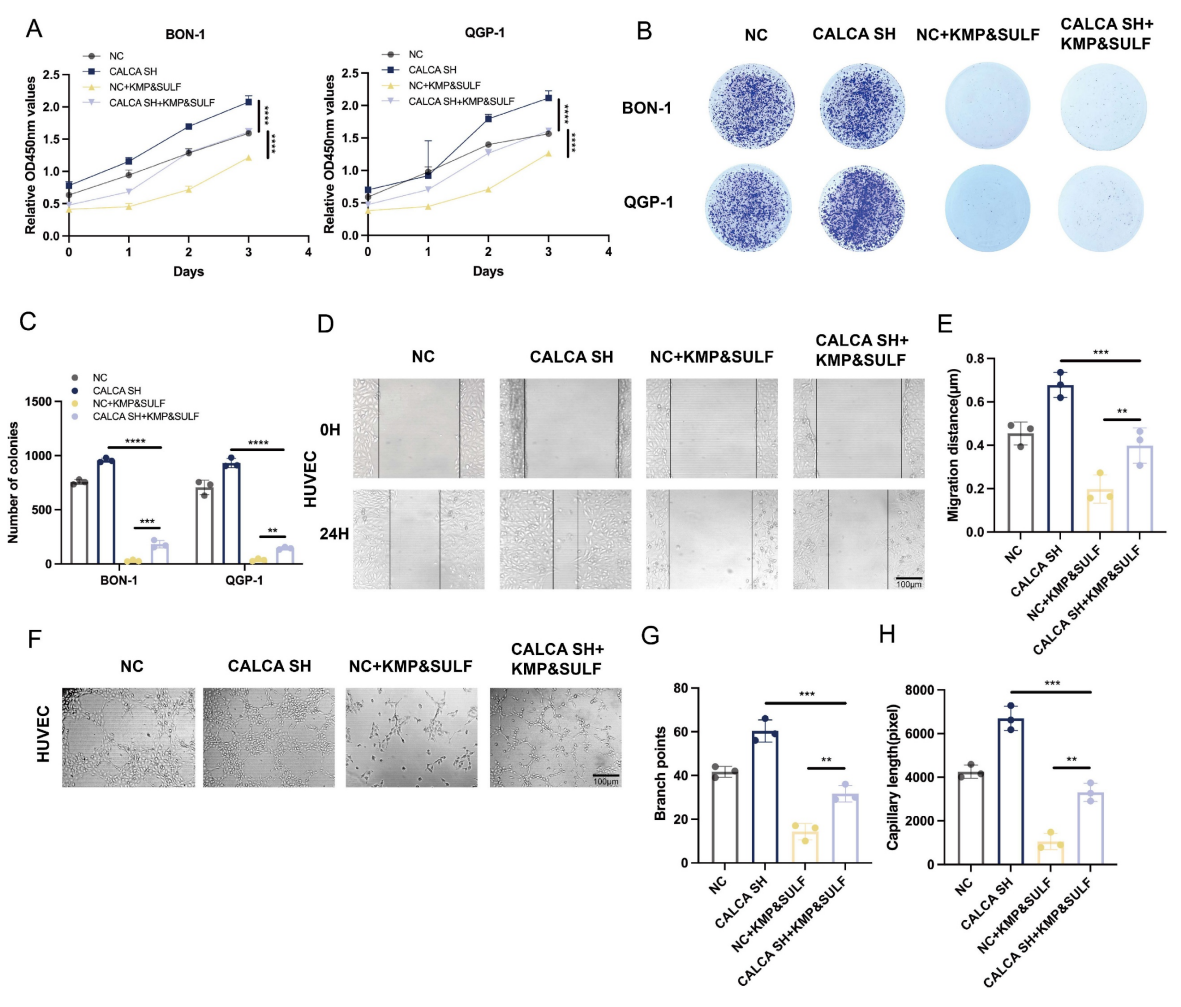
Drug combination inhibits CALCA knockdown-induced malignant behavior in pNET cells. (A-H) CCK-8, colony formation, wound healing assay, and tubule formation assay showing cell proliferation under the effect of CALCA knockdown and drug combination treatment. (Data were shown as mean±SD. n=3. Statistical significance between groups was determined by one-way ANOVA). Scale bar = 100μm.*p < 0.05, **p < 0.01, ***p < 0.001, ****p < 0.0001, ns: no significance.

The results from the CCK-8 assay indicated that, relative to the untreated knockdown cohort, the combination therapy significantly reduced the proliferative capacity of tumor cells resulting from CALCA depletion. Moreover, CALCA knockdown undermined the tumor-suppressive impact of the combination when compared to the combination-only group (Fig. 7A). The colony formation assay revealed that the increased clonogenic potential triggered by CALCA knockdown was effectively diminished by the combination therapy (Fig. 7B,C). Additionally, the combination was successful in curtailing the pro-angiogenic effects and the migration tendencies of vascular endothelial cells induced by CALCA knockdown. The wound healing assay illustrated that HUVECs exposed to the CALCA knockdown conditioned medium demonstrated enhanced tumor cell migration, an effect that was subsequently reversed upon treatment with the combination (Fig. 7D,E). The angiogenesis assay further confirmed that the number of vascular branching points and the length of vessel formation were notably elevated in CALCA knockout cells. However, this heightened tube formation was effectively negated by the combination treatment (Fig. 7F-H). These findings suggest that the Sulfatinib-Kaempferol therapeutic regimen is proficient in counteracting the proliferation, migration, and angiogenesis induced by CALCA knockdown in pNETs cells. The effectiveness of this combination therapy indicates its potential to target CALCA-related pathways or their downstream mediators that are vital for tumor advancement, thereby underscoring the prospective clinical significance of this combinatorial approach for patients suffering from CALCA-deficient neuroendocrine tumors.

### Knockdown of CALCA restores tumor suppression by Sulfatinib and Kaempferol combination

**Figure 8:**
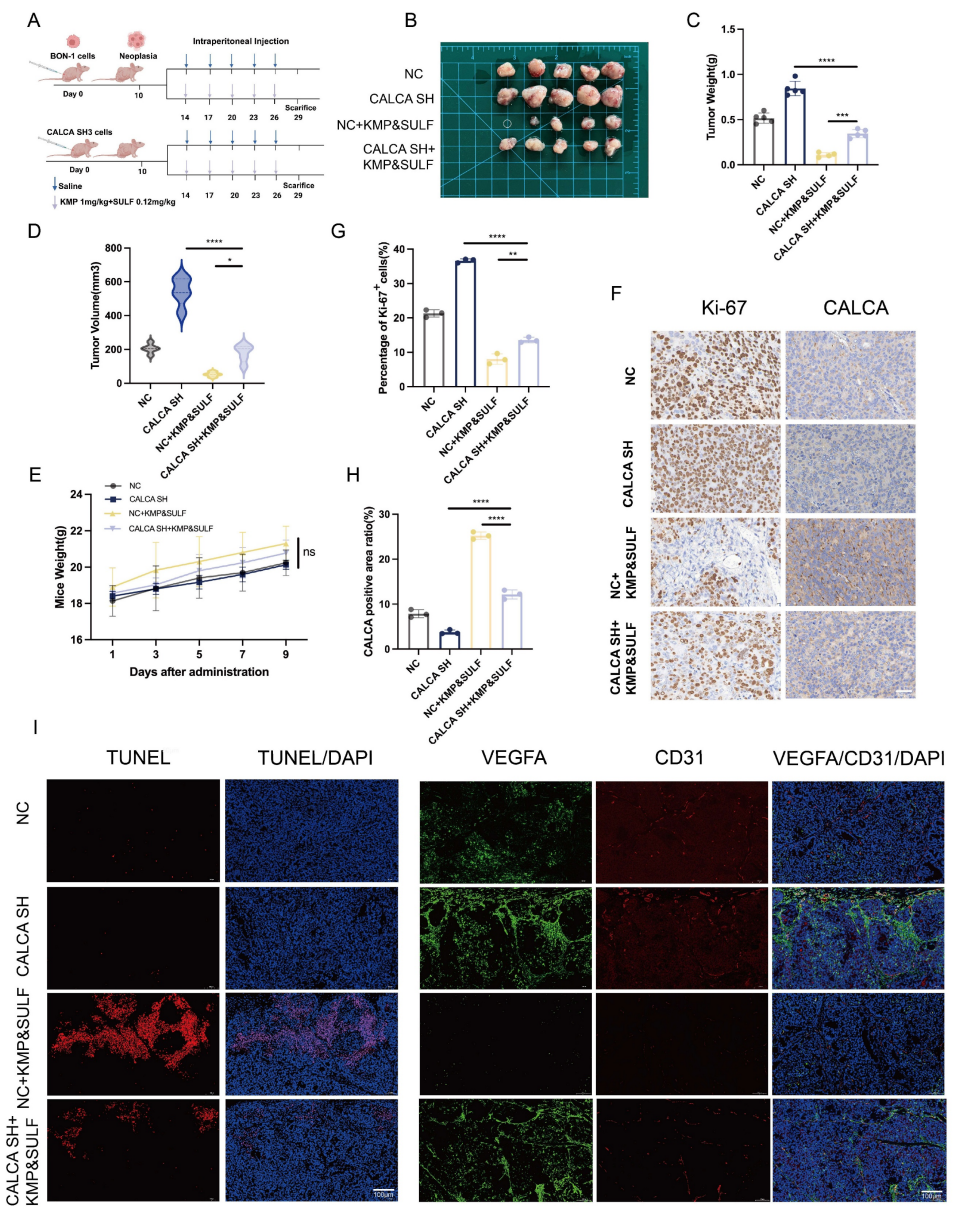
Knockdown of CALCA restores tumor suppression by drug combination *in vivo*. (A-E) Primary tumor samples obtained from nude mice after subcutaneous injection of BON-1 cells in the control, CALCA knockdown, co-drug and CALCA knockdown plus co-drug treatment groups. (A-B) Schematic of the subcutaneous tumor inoculation model and representative tumor images in nude mice. (C)Relative tumor weight, (D)tumor volume and (E) nude mouse body weight was measured at the endpoint. (Data were shown as mean±SD. n=5. Statistical significance between groups was determined by one-way ANOVA). (F-H) Representative immunohistochemical images of Ki-67 and CALCA expression in xenograft tumor tissues and the corresponding quantitative analysis. (Data were shown as mean±SD. n=3. Statistical significance between groups was determined by one-way ANOVA). Scale bar = 50μm. (I)The following tests were performed on tumor tissues from each group: immunofluorescence, apoptosis staining, TUNEL staining, angiogenesis-related staining, VEGFA staining and CD31 staining. Scale bar = 100μm. *p < 0.05, **p < 0.01, ***p < 0.001, ****p < 0.0001, ns: no significance.

In order to evaluate the efficacy of Sulfatinib and Kaempferol in counteracting the aggressive characteristics associated with CALCA knockdown, a xenograft model was developed utilizing BON-1 cells, both with and without CALCA knockdown. The progression of tumors under the combined treatment was closely observed (Fig. 8A,B). The results indicated that the group subjected to the combination treatment following CALCA knockdown displayed an accelerated tumor growth rate when compared to the group receiving only the combination treatment. Notably, however, both tumor volume and weight were significantly higher in the knockdown group in comparison to the latter. The combination of the two pharmacological agents effectively inhibited tumor proliferation instigated by CALCA knockdown (Fig. 8C,D). Additionally, no considerable changes in the weight of the nude mice were recorded across the various experimental groups (Fig. 8E). IHC evaluations revealed a marked reduction in the levels of Ki-67 in the knockdown group treated with the drug combination, when juxtaposed with the knockdown group alone, thus confirming the retained efficacy of both agents. Throughout the study, the combination treatment group consistently demonstrated suppressed tumor growth along with high CALCA expression (Fig. 8F-H). IF demonstrated that the combination therapy reversed the anti-apoptotic effect of CALCA knockdown, significantly enhancing apoptosis compared to the knockdown-alone group. This observation emphasizes the inhibitory effect of the drug combination on pNETs growth. Similarly, compared to the knockdown-alone group, the combination therapy significantly reduced angiogenesis in the knockdown group, along with decreased expression levels of VEGFA and CD31 (Fig. 8I). These *in vivo* results suggest that the combination of Sulfatinib and Kaempferol preserves its therapeutic effectiveness in tumors deficient in CALCA, thereby offering critical preclinical insights for targeting aggressive neuroendocrine tumors characterized by CALCA genetic alterations.

### Combination of Sulfatinib and Kaempferol inhibits pNETs progression via inactivating PI3K/AKT/mTOR pathway

**Figure 9:**
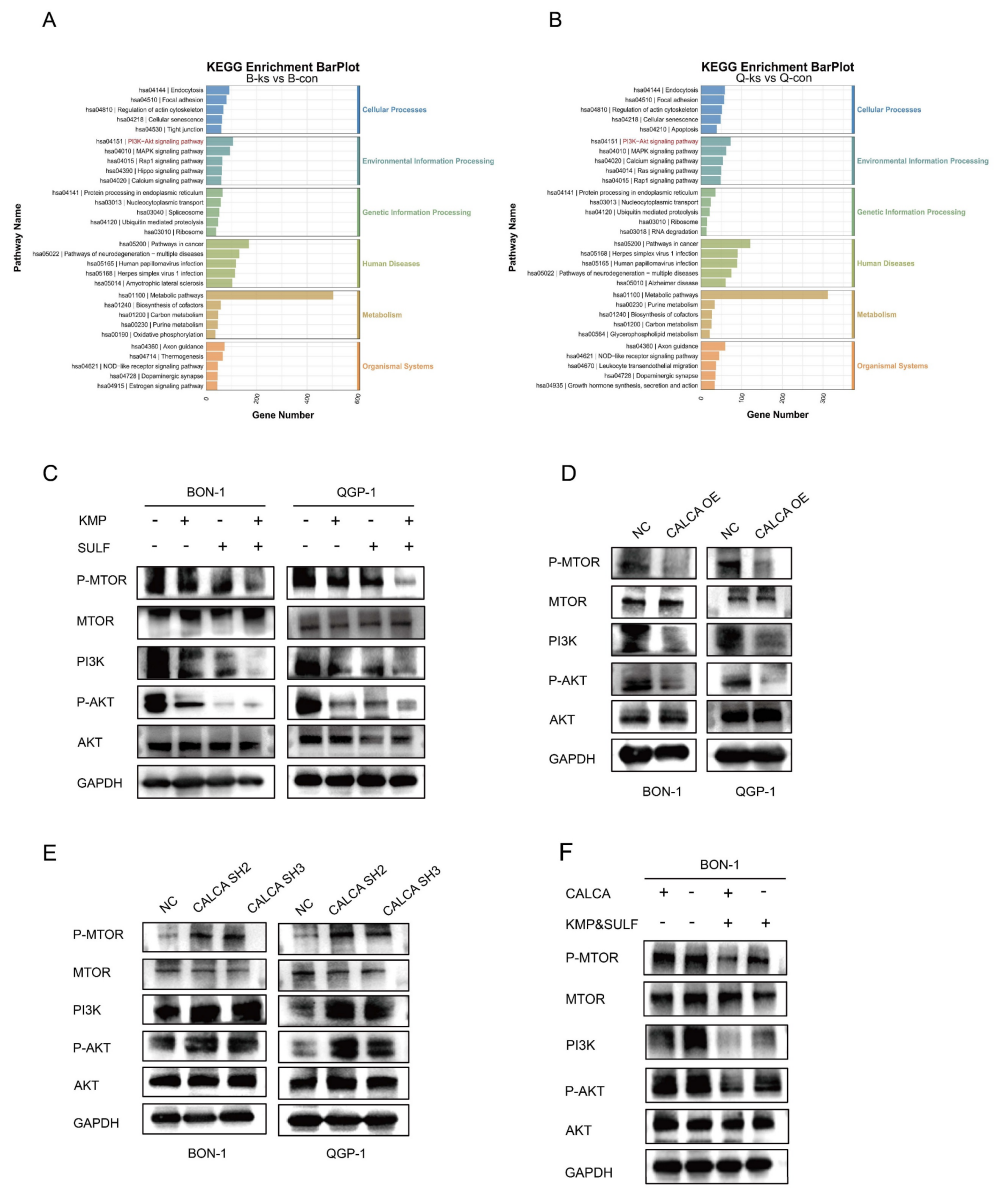
The combination of the two drugs was used to inhibit neuroendocrine tumor progression in a PI3K/AKT/mTOR pathway-dependent pathway. (A,B) Analysis of KEGG enrichment of different genes that were induced by CON in comparison with the combination of BON-1 or QGP-1. (C)Western blotting results of PI3K/AKT/mTOR signaling pathway proteins expression in BON-1 and QGP-1 cells 24 hours after treatment with PBS, Kaempferol, Sulfatinib, and the combination therapy. (D,E) Western blotting assay to evaluate the expression levels of genes associated with the PI3K/AKT/mTOR signaling pathway in BON-1 and QGP-1 cells. These cells had been transiently transformed with NC Vector, CALCA overexpression plasmid, and CALCA knockdown plasmid. (F) Western blotting to detect the expression of PI3K/AKT/mTOR signaling pathway related genes in BON-1 cells transfected with NC Vector, CALCA knockdown plasmid, NC co-drug group and CALCA knockdown plasmid co-drug group.

To clarify the molecular mechanisms underlying the synergistic anti-tumor effects of Sulfatinib and Kaempferol, a study was undertaken to assess their impact on the PI3K/AKT/mTOR signaling pathway within neuroendocrine tumor cells. KEGG pathway analysis indicated that the PI3K/AKT/mTOR signaling pathway emerged as one of the most significantly modified pathways in BON-1 and QGP-1 cells following combination treatment, potentially elucidating the mechanism by which these two agents collectively suppress tumor proliferation (Fig. 9A,B). To further investigate this proposition, Western blot analysis was employed to assess the status of the PI3K/AKT/mTOR pathway. The results corroborated those cells subjected to the combined treatment exhibited a suppression of the PI3K/AKT/mTOR signaling pathway (Fig. 9C). Additional examinations indicated that the expression of CALCA played a pivotal role in mediating the inhibition of the PI3K/AKT/mTOR pathway (Fig. 9D), while the silencing of CALCA led to the pathway's activation (Fig. 9E). Notably, the administration of the drugs was found to counteract the activation effect prompted by CALCA knockdown (Fig. 9F). These findings suggest that the antitumor efficacy of the Sulfatinib and Kaempferol combination is achieved through a dual inhibition mechanism of the PI3K/AKT/mTOR signaling pathway, alongside functional interactions with CALCA, a principal regulator within the pathway, thereby synergistically thwarting tumor development. The intricate relationships among gene expression regulation, metabolic adaptations, and signaling activation were highlighted, providing fresh perspectives on potential therapeutic targets.

## Discussion

Sulfatinib is an important therapeutic option for advanced pNETs, as it inhibits angiogenesis and modulates the tumor immune microenvironment by targeting VEGFR, FGFR and CSF-1R^15^, ^23^, ^24^. Sulfatinib is approved in China for monotherapy in the treatment of unresectable, locally advanced, or metastatic neuroendocrine tumors^12^, ^25^. However, clinical use of Sulfatinib is associated with acquired resistance and adverse effects, including hypertension, proteinuria and potential hepatotoxicity and nephrotoxicity. These effects may require dose adjustment or discontinuation of the drug, resulting in diminished antitumor efficacy^14^, ^23^. In light of these challenges, there is an urgent need to develop personalized, safer treatment strategies for pNETs. A previous phase II clinical study demonstrated the potential for Sulfatinib to have a synergistic anti-tumor effect when combined with Toripalimab, an immune checkpoint inhibitor, in patients with advanced neuroendocrine tumors and neuroendocrine carcinomas. The study also showed that this combination had a favorable safety profile^26^. These results provide a theoretical basis for subsequent experimental validation. Future studies should focus on optimizing therapeutic regimens to reduce toxicity and overcome drug resistance, thereby improving the long-term prognosis of patients with pNETs.

In this study, we attempted to enhance the sensitivity of pNETs cells to Sulfatinib receptor agonists by using Kaempferol to achieve synergistic effects at concentrations below the IC₅₀, thereby enabling combination therapy with superior vascular targeting ability and a higher safety profile. However, the combined anti-tumor activity of the two agents and their mechanism of action on pNETs remain unknown. Therefore, we conducted this study and drew four main conclusions. Firstly, we found that the combination of Sulfatinib and Kaempferol significantly reduced proliferation and angiogenesis, and promoted apoptosis in pNETs cells. Secondly, the combination of the two drugs significantly reduced tumor weight and volume in nude mice compared to Sulfatinib or Kaempferol monotherapy. Thirdly, transcriptomic results indicated that the combination could inhibit pNETs progression by downregulating the PI3K/AKT/mTOR pathway and overexpressing the CALCA gene. Fourthly, the two-drug combination reversed the effects of CALCA knockdown on cell proliferation and angiogenesis in pNETs by downregulating the PI3K/AKT/mTOR signaling pathway. This study demonstrated that Kaempferol acts as a sensitizer of Sulfatinib and exerts a synergistic effect when used in combination, thereby improving the efficacy and safety of clinical treatment.

Previous studies have shown that Kaempferol, a natural flavonoid found in various plants and plant-derived foods such as tomatoes, broccoli and apples, has a wide range of antitumor activities that provide new ideas and possibilities for tumor therapy^27^-^29^. Similar to other polyphenolic compounds, Kaempferol acts as an antioxidant and can help extend food shelf life^30^, ^31^. Additionally, due to its anti-glycemic properties, Kaempferol derivatives can be used to prevent and treat diabetes^32^, ^33^. This flavonoid regulates proteins related to apoptosis, angiogenesis, inflammation, and metastasis, inhibits the PI3K/AKT/mTOR pathway and indirectly prevents tumor cells migration, thus exerting its antitumor effects^7^, ^20^, ^34^. Kaempferol has been demonstrated to exhibit potent antitumor effects in hepatocellular carcinoma, colorectal carcinoma, non-small-cell lung carcinoma, and pancreatic carcinoma, both *in vitro* and *in vivo*^16^, ^17^, ^35^-^37^. In addition, Kaempferol has been found to synergies with a variety of chemotherapeutic agents. When used in combination with etoposide, Kaempferol enhances the sensitivity of granulocytes to etoposide and reduces the generation of free radicals^19^. The combination of Kaempferol and Docetaxel has also been shown to be effective in prostate cancer cells. Application of the combination also showed good anti-tumor activity in prostate cancer cells, inducing autophagy and enhancing sensitivity to docetaxel while reducing drug resistance during chemotherapy^38^. Kaempferol, when used in combination with cisplatin, inhibited cell migration by inducing apoptosis and increasing sensitivity to cisplatin in head and neck cancer cells^39^. In addition, Kaempferol has been found to inhibit the migration and invasion of tumor cells, thereby preventing the spread of cancer cells and tumor metastasis. This finding suggests that Kaempferol could be used alongside chemotherapy to improve its efficacy. However, the anticancer mechanism of Kaempferol in NETs is still unclear.

Our current study demonstrates that Sulfatinib and Kaempferol have synergistic effects in pNETs. *In vitro*, it was shown that the combination of Sulfatinib and Kaempferol significantly inhibited the proliferation and angiogenesis of pNETs, based on the optimal drug combination concentration (Fig. 1C), CCK-8 (Fig. 2A), colony formation (Fig. 2B, C) and angiogenesis (Fig. 2H, I) assays. Our results showed that the KMP concentration of 100 µM contained the highest synergistic region at the lowest concentration. Compared to the IC₅₀ of KMP (BON-1: 144.3 µM; QGP-1: 138.4 µM) and the concentrations of KMP previously used in other tumor model studies, we employed a dose of 100 µM on a significantly reduced effective basis^16^. In the xenograft model (Fig. 3), mice were administered 0.12 mg/kg of SULF and/or 1 mg/kg of KMP intraperitoneally every two days. After two weeks, we observed that the tumor volume and weight of the mice treated with combination therapy were much smaller than those of the mice in the other three groups (saline control, SULF alone and KMP alone). These results suggest that Kaempferol is effective and safe in enhancing Sulfatinib treatment in pNETs. Additionally, we observed a significant increase in apoptosis in pNETs cells treated with SULF and KMP, as determined by flow analysis (Fig. 2F, G) and TUNEL staining (Fig. 3I), particularly in the late apoptotic stage.

We performed transcriptomics on the control and co-drug groups of both cell lines and identified 14 genes associated with angiogenesis (Fig. 4E). Of these, six were up-regulated (ITLN1, TNFSF15, CGA, CALCA, MIAT and MEG3) and eight were down-regulated (ROR2, SERPINA1, BMP7, S100A4, IGF2, HNF4A, SEMA3F and SST). We analyzed the 14 genes by qPCR and found that CALCA differed most significantly. Studies have shown that calcitonin gene-related peptide (CGRP) has a variety of biological functions and that downregulation of its expression level can promote tumor progression by enhancing angiogenesis^40^, ^41^. CALCA is one of the CGRP isoforms and plays a diverse and complex role in chronic low-grade inflammation. It is also closely associated with specific cancers. CALCA has been reported to play a key role in regulating apoptosis and oxidative stress through the PI3K/Akt pathway^29^. Therefore, we hypothesized that CALCA induces apoptosis and inhibits angiogenesis in the pathogenesis of pNETs. However, the mechanism of action of CALCA in pNETs remains unclear. In this study, we performed IHC staining for CALCA in human pancreatic cancer and paracancerous tissues. The result showed that pancreatic cancer expressed low levels of CALCA, while paracancerous tissues expressed high levels (Fig. 4K). This was verified by CALCA knockdown and overexpression models. We concluded that CALCA acts as a tumor suppressor in pNETs, inhibiting cell proliferation and angiogenesis by downregulating the PI3K/AKT/mTOR signaling pathway. It is worth noting that in the rescue experiment, we treated CALCA-overexpressed pNETs cells with the mTOR agonist MHY1485, which could significantly reverse the proliferation inhibition induced by CALCA overexpression and promote tumor growth and angiogenesis. This discovery has been consistently verified in both *in vivo* and *in vitro* experiments. This provides evidence for the PI3K/AKT/mTOR pathway as a key downstream mechanism of the synergistic effect between Sulfatinib and Kaempferol, with CALCA located upstream of this pathway and playing a regulatory role. However, this study still has some limitations. Our *in vivo* experiments mainly rely on subcutaneous xenograft models, which lack the microenvironment of the primary tumor and are difficult to effectively simulate the metastasis process of pNETs. In future research, we will focus on establishing a liver metastasis model to further verify the synergistic effect of Sulfatinib and Kaempferol in inhibiting tumor invasion and metastasis, and to deeply explore its potential molecular mechanisms.

In conclusion, we demonstrated that Sulfatinib and Kaempferol inhibited the proliferation and angiogenesis of pNETs *in vivo* and *in vitro* by downregulating the PI3K/AKT/mTOR signaling pathway, increasing CALCA expression and inducing apoptosis in pNETs synergistically. These findings suggest that CALCA could serve as a therapeutic target or biomarker for pNETs. Further studies will seek to clarify the molecular mechanisms underlying the cooperative interactions between Sulfatinib and Kaempferol in pNETs, thereby strengthening the theoretical basis for developing combination therapies and anti-angiogenic drugs.

## Supplementary Material

Supplementary figures and tables.

## Abbreviations

PNETs: Pancreatic neuroendocrine tumors
GEP-NETs: Gastroenteropancreatic neuroendocrine tumors
SULF: Sulfatinib
KMP: Kaempferol
NETs: Neuroendocrine tumors
VEGF: Vascular endothelial growth factor
VEGFR: Vascular endothelial growth factor receptor
FGFR1: Fibroblast growth factor 1
CSF1R: Colony-stimulating factor 1 receptor
IC50: Half-maximal inhibitory concentration
CCK-8: Cell counting kit-8
HUVECs: Human umbilical vein endothelial cells
EdU: 5-Ethynyl-2'-deoxyuridine
WB: Western blotting
qRT-PCR: Quantitative real-time polymerase chain reaction
IHC: Immunohistochemical
IF: Immunofluorescence
CGRP: Calcitonin gene-related peptide
