# Supplementary material for: Synergistic Anti-Tumor Effects of Sulfatinib and Kaempferol on Pancreatic Neuroendocrine Tumors via CALCA-mediated PI3K/AKT/mTOR Pathway
Source: Int J Biol Sci. 2025 Oct 27;21(15):7043–62. doi: 10.7150/ijbs.119176 (PMC12631220; doi:10.7150/ijbs.119176)
Supplement: Supplementary file 1 — Supplementary figures and tables. [file ijbsv21p7043s1.pdf]

Supplementary Materials

Supplementary Figures

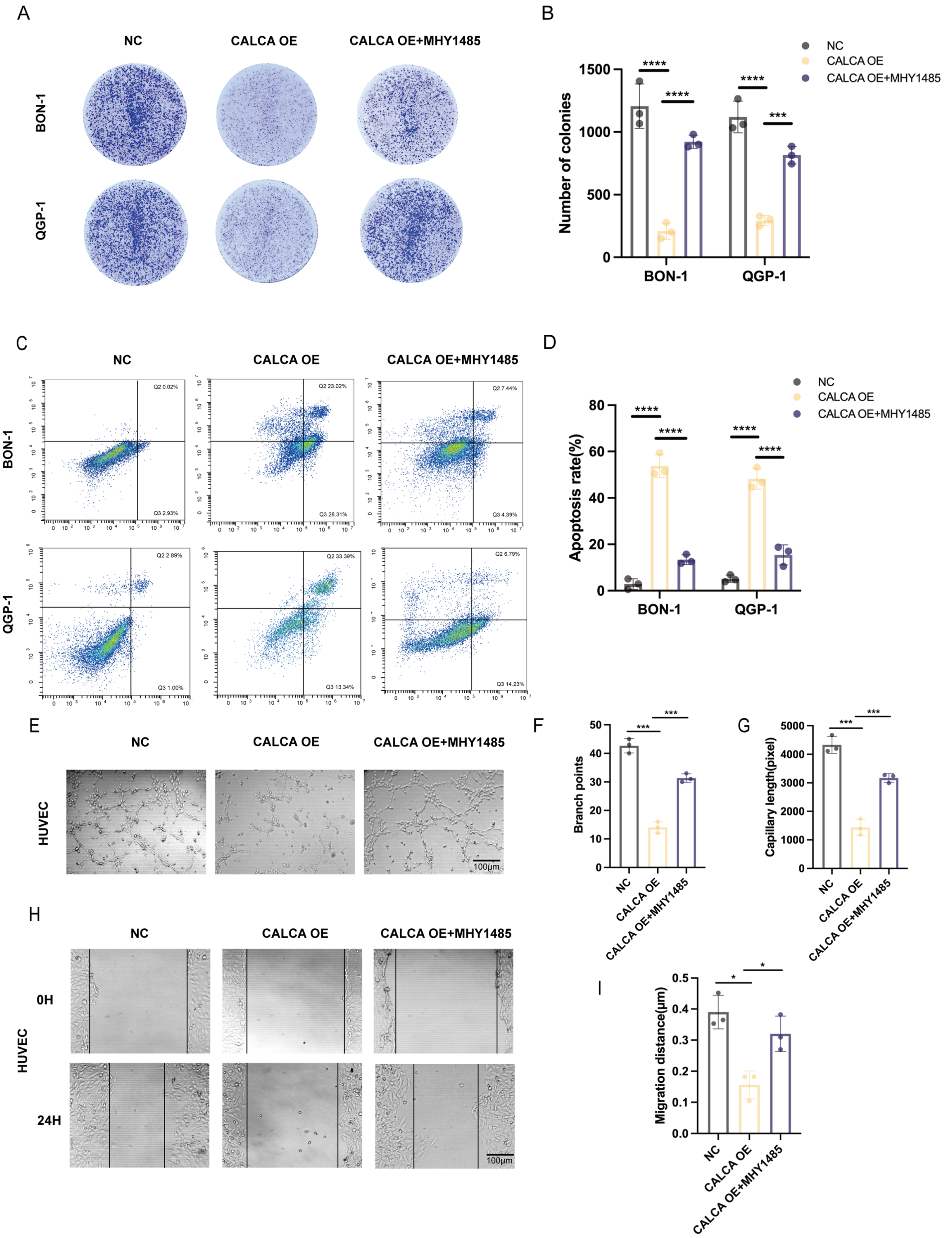

Figure S1: MHY1485 reverses the tumor-suppressive effects of CALCA overexpression. (A,B) Colony formation assay assessing proliferation in BON-1 and QGP-1 cells treated with CALCA OE and CALCA OE + MHY1485 for 24 hours(Data were shown as mean±SD. n=3. Statistical significance between groups was determined by two-way ANOVA).(C,D) Flow cytometry analysis with PI/Annexin-V staining to evaluate cell viability after adding MHY1485 to CALCA OE cells(Data were shown as mean±SD. n=3. Statistical significance between groups was determined by two-way ANOVA).(E-G) In vitro tube formation assay performed on NC, CALCA

OE, and CALCA OE + MHY1485 groups. (Data were shown as mean±SD. n=3. Statistical significance between groups was determined by one-way ANOVA). Scale bar=100μm. (H,I) Wound healing assay of HUVEC cells co-cultured with CALCA OE and CALCA OE + MHY1485 pNETs cells for 24 hours. (Data were shown as mean±SD. n=3. Statistical significance between groups was determined by one-way ANOVA). Scale bar=100μm. $*p < 0.05$, $**p < 0.01$, $***p < 0.001$, $****p < 0.0001$, ns: no significance.

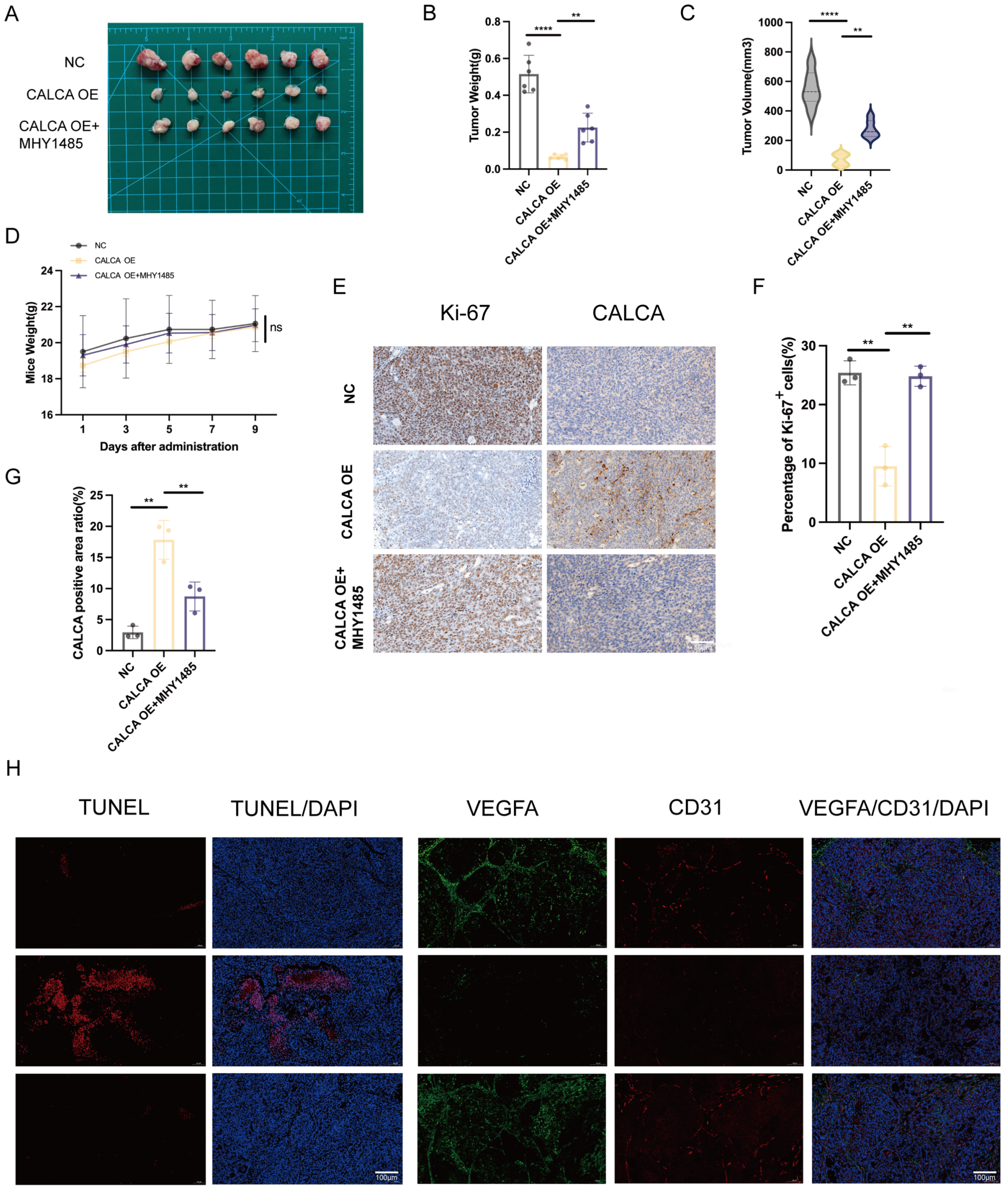

Figure S2: The inhibitory effect of CALCA overexpression in vivo on tumor growth was reversed by MHY1485. (A-D) Primary tumor samples were obtained from nude mice in the control, CALCA overexpression, and CALCA overexpression + MHY1485 groups after subcutaneous

injection of BON-1 cells. Images of subcutaneous tumors in nude mice(A).Relative tumor weight (B), tumor volume (C) and nude mouse body weight (D)were measured at the endpoint.(Data were shown as mean±SD. n=6. Statistical significance between groups was determined by one-way ANOVA).(E-G)Representative immunohistochemical images showing Ki-67 and CALCA expression in xenograft tumor tissues, with quantitative analysis.(Data were shown as mean±SD. n=3. Statistical significance between groups was determined by one-way ANOVA). Scale bar=50μm. (H)The following tests were performed on tumor tissues from each group: immunofluorescence, apoptosis staining, TUNEL staining, angiogenesis-related staining, VEGFA staining and CD31 staining. Scale bar=100μm.*p < 0.05, **p < 0.01, ***p < 0.001, ****p < 0.0001, ns: no significance.

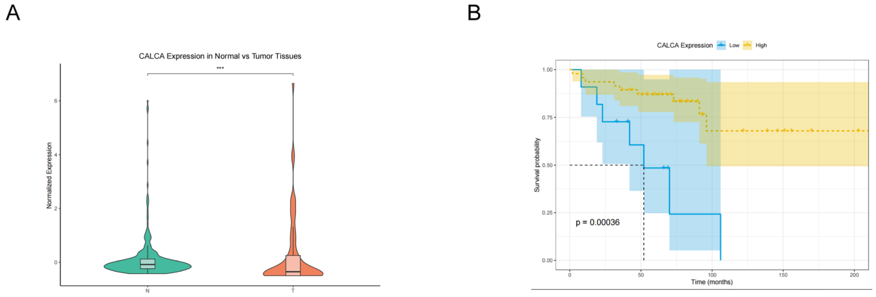

FigureS3: Expression and Prognostic Analysis of CALCA. (A)Relative expression levels of CALCA in Gene Expression Omnibus (GEO; accession GSE98894) and the Genotype-Tissue Expression (GTEx) project (Normal: 357cases; Tumor: 67cases). (B) ROC curve analysis of CALCA in pancreatic neuroendocrine neoplasm using European Genome-phenome Archive (EGA; accession EGAS00001005024). (n=84).

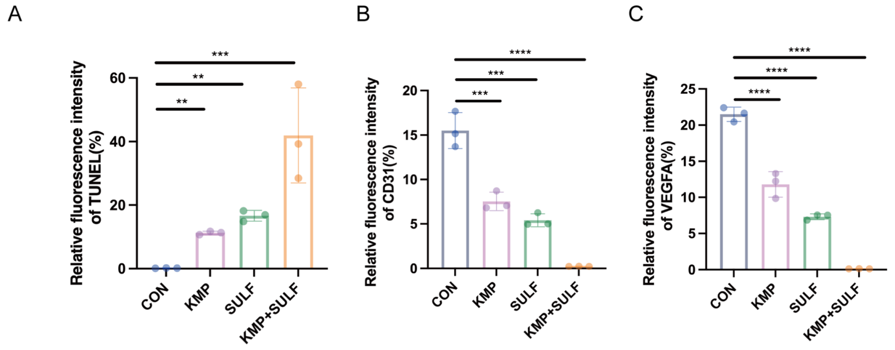

FigureS4: The combination of drugs can enhance the inhibitory effect on tumor angiogenesis in vivo. (A-C)Quantitative analysis of tumor tissue immunofluorescence apoptosis staining TUNEL and angiogenesis-related staining VEGFA and CD31 in the combination drug group. (Data were shown as mean±SD. n=3. Statistical significance between groups was determined by one-way

ANOVA). *p < 0.05, **p < 0.01, ***p < 0.001, ****p < 0.0001, ns: no significance.

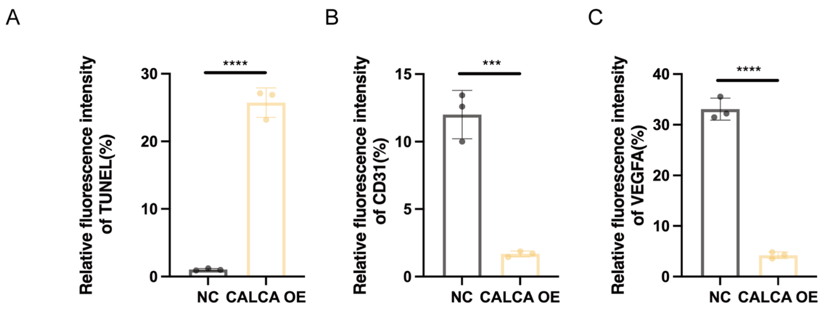

FigureS5: The overexpression of CALCA can potentiate the anti-angiogenic effects on tumor vascularization in vivo. (A-C)Quantitative analysis of tumor tissue immunofluorescence apoptosis staining TUNEL and angiogenesis-related staining VEGFA and CD31 in CALCA-overexpressing tumor tissues. (Data were shown as mean±SD. n=3. Statistical analysis was performed using a two-tailed unpaired Student's t-test). *p < 0.05, **p < 0.01, ***p < 0.001, ****p < 0.0001, ns: no significance.

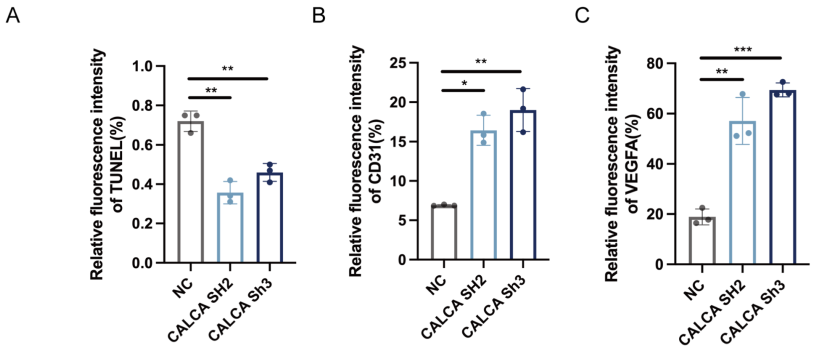

FigureS6: Knockdown of CALCA can enhance tumor growth and angiogenesis in vivo.(A-C)Quantitative analysis was conducted on the immunofluorescence apoptosis staining TUNEL and angiogenesis-related staining VEGFA and CD31 in tumor tissues with CALCA knockdown. (Data were shown as mean±SD. n=3. Statistical significance between groups was determined by one-way ANOVA). *p < 0.05, **p < 0.01, ***p < 0.001, ****p < 0.0001, ns: no significance.

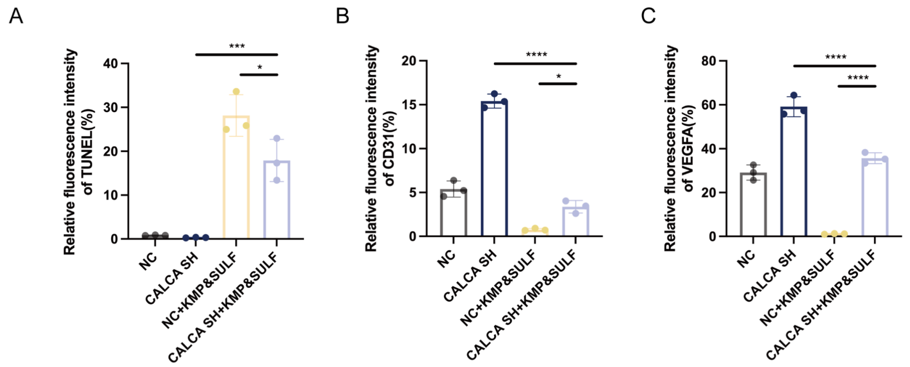

FigureS7: Knocking down CALCA can restore the inhibitory effect of drug combination on angiogenesis in vivo. (A-C)Quantitative analysis of immunofluorescence apoptosis staining using TUNEL and angiogenesis-associated markers VEGFA and CD31 across various tumor tissue groups. (Data were shown as mean±SD. n=3. Statistical significance between groups was determined by one-way ANOVA). *p < 0.05, **p < 0.01, ***p < 0.001, ****p < 0.0001, ns: no significance.

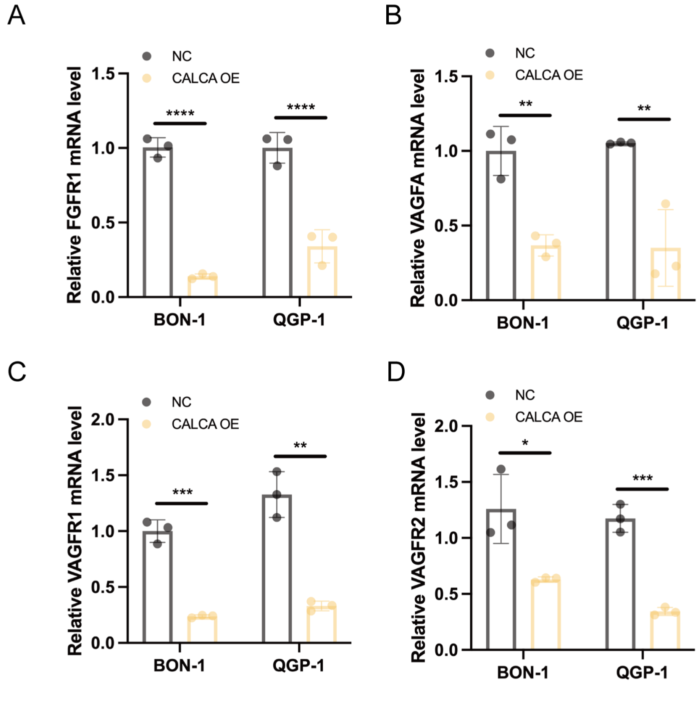

FigureS8: mRNA expression of angiogenesis-related genes in the CALCA OE group. (A-D)Quantitative reverse transcription polymerase chain reaction (qRT-PCR) was employed to

quantify the mRNA expression levels of FGFR1, VEGFA, VEGFR1, and VEGFR2 in BON-1 and QGP-1 neuroendocrine tumor cells. (Data were shown as mean±SD. n=3. Statistical significance between groups was determined by two-way ANOVA). *$p < 0.05$, **$p < 0.01$, ***$p < 0.001$, ****$p < 0.0001$, ns: no significance.

Supplementary Tables

Table S1 Antibody information

Antibody	Company	Catalogue	Dilution ratio
FGFR1	Proteintech	60325-1-Ig	1：1000
VEGFA	Proteintech	81323-2-RR	1：1000
VEGFR1	Origene	TA384238	1：1000
VEGFR2	Abcam	ab134191	1：1000
CALCA	Proteintech	68774-1-Ig	1：1000
KI-67	Proteintech	84192-4-RR	1：1000
GAPDH	Proteintech	60004-1-Ig	1：5000
mTOR	Proteintech	66888-1-Ig	1：1000
Phospho-mTOR	Proteintech	67778-1-Ig	1：1000
PI3 Kinase	Proteintech	20584-1-AP	1：1000
AKT	Proteintech	10176-2-AP	1：1000
Phospho-AKT	Proteintech	66444-1-Ig	1：1000
Goat Anti-Mouse IgG	CWBIO	CW0102S	1：5000
Goat Anti-Rabbit IgG	CWBIO	CW0103S	1：2000

Table S2 Sequences of RT-qPCR primers

Gene names	Sequence(5’-3’)
ITLN1 forward	ACGTGCCCAATAAGTCCCC
ITLN1 reverse	CCGTTGTCAGTCCAACACTTTC
ROR2 forward	TCCGAACGACCCTTTAGGAC
ROR2 reverse	TTTAGCCACCGCACGTTAGG
TNFSF15 forward	GCACCTCTTAGAGCAGACGG
TNFSF15 reverse	CGGAATGTGACCTGGGAGTAAAT
CGA forward	ATGATGCTGAATGTGCGGAAT
CGA reverse	CGCCAACTGGATAAATTGGACTT
CALCA forward	GCTTGAAAACAAGGGCCAAGT
CALCA reverse	GGTTGCTCTGCTCAACAGAAAA
SERPINA1 forward	TGAGGCACGATGGCAACAT
SERPINA1 reverse	GAGCCCTCTCTTGATCTGGG
BMP7 forward	TCGGCACCCATGTTCATGC
BMP7 reverse	GAGGAAATGGCTATCTTGCAGG
MIAT forward	CACTGCTCCTGGATTCTGTTCTTGG
MIAT reverse	AACGCTGGGACTGTCTCCTCTG
S100A4 forward	GATGAGCAACTTGGACAGCAA
S100A4 reverse	CTGGGCTGCTTATCTGGGAAG
IGF2 forward	TATATCGGAAACCTCAGCGAGA

IGF2 reverse	GGACCGAGTGCTCAACTTCT
HNF4A forward	CACGGGCAAACACTACGGT
HNF4A reverse	TTGACCTTCGAGTGCTGATCC
MEG3 forward	CCTCACCTCCAATTTCCTCTTC
MEG3 reverse	TCCAGCAGCTAACCTCATTAAC
SEMA3F forward	AACACAACCGACTACCGAATC
SEMA3F reverse	GGCTGCCCAGTGTATAATGAG
SST forward	ACCCAACCAGACGGAGAATGA
SST reverse	GCCGGGTTTGAGTTAGCAGA
FGFR1 forward	ACCAAACCGTATGCCCGTAG
FGFR1 reverse	CAGGTGGCATAACGGACCTT
VEGFA forward	ACATCACCATGCAGATTATGCG
VEGFA reverse	CTCCAGGGCATTAGACAGCA
VEGFR1 forward	TTCCGAAGCAAGGTGTGACT
VEGFR1 reverse	AGAGTCAGCCACAACCAAGG
VEGFR2 forward	GAGGGGAACTGAAGACAGGC
VEGFR2 reverse	GGCCAAGAGGCTTACCTAGC
GAPDH forward	GGAGCGAGATCCCTCCAAAAT
GAPDH reverse	GGCTGTTGTCATACTTCTCATGG
